# The Impact of Normalization Approaches to Automatically Detect Radiogenomic Phenotypes Characterizing Breast Cancer Receptors Status

**DOI:** 10.3390/cancers12020518

**Published:** 2020-02-24

**Authors:** Rossana Castaldo, Katia Pane, Emanuele Nicolai, Marco Salvatore, Monica Franzese

**Affiliations:** IRCCS SDN, Via E. Gianturco, 113, 80143 Naples, Italy; rcastaldo@sdn-napoli.it (R.C.); enicolai@sdn-napoli.it (E.N.); direzionescientifica@sdn-napoli.it (M.S.); mfranzese@sdn-napoli.it (M.F.)

**Keywords:** Molecular imaging, breast cancer, miRNA expression, radiogenomics, machine learning, radiomic, diagnosis, biomarker

## Abstract

In breast cancer studies, combining quantitative radiomic with genomic signatures can help identifying and characterizing radiogenomic phenotypes, in function of molecular receptor status. Biomedical imaging processing lacks standards in radiomic feature normalization methods and neglecting feature normalization can highly bias the overall analysis. This study evaluates the effect of several normalization techniques to predict four clinical phenotypes such as estrogen receptor (ER), progesterone receptor (PR), human epidermal growth factor receptor 2 (HER2), and triple negative (TN) status, by quantitative features. The Cancer Imaging Archive (TCIA) radiomic features from 91 T1-weighted Dynamic Contrast Enhancement MRI of invasive breast cancers were investigated in association with breast invasive carcinoma miRNA expression profiling from the Cancer Genome Atlas (TCGA). Three advanced machine learning techniques (Support Vector Machine, Random Forest, and Naïve Bayesian) were investigated to distinguish between molecular prognostic indicators and achieved an area under the ROC curve (AUC) values of 86%, 93%, 91%, and 91% for the prediction of ER+ versus ER−, PR+ versus PR−, HER2+ versus HER2−, and triple-negative, respectively. In conclusion, radiomic features enable to discriminate major breast cancer molecular subtypes and may yield a potential imaging biomarker for advancing precision medicine.

## 1. Introduction

Breast cancer is the most frequently diagnosed cancer among women, and it is the second leading cause of death in women [1]. Based on the molecular receptor status, breast cancer can be classified into different subtypes with different response to therapy and prognosis. The three clinically most-useful receptors status to characterize breast cancer cells are the estrogen receptor (ER), progesterone receptor (PR), and human epidermal growth factor receptor 2 (HER2) that can impact therapy and prognosis [2]. 

Breast cancer is a heterogeneous disease. Indeed, HER2-positive (HER2+) breast cancers are more aggressive and show a poorer prognosis than HER2-negative (HER2–) cancers. Positive hormonal receptor status such as ER-positive (ER+) and PR-positive (PR+) tumor have lower risks of mortality than ER-negative (ER–) and/or PR-negative (PR–) disease [3,4,5,6,7]. Triple negative (TN) tumor (negative for all three receptors) shows a high relapsing rate, and therefore, accounts for a large portion of breast cancer deaths. Therefore, it becomes necessary to identify molecular receptor status and subsequently subtypes to select the appropriate therapy and predict the therapeutic response [8,9].

Radiomics has recently emerged as a promising tool for discovering non-invasive imaging signatures characterizing lesions such as size, shape, descriptors of the image intensity histogram and texture [10,11]. The term ‘Radiomics’ refers to the high-throughput extraction of quantitative features from medical images, i.e., conversion of images to mineable data, that can potentially capture tumor heterogeneity, aiding precision medicine [12]. A closely related field is ‘radiogenomics’, which explores the associations between imaging phenotype (radiomic data) and disease genotype (genomic patterns) [13,14]. Two public data resources such as The Cancer Genome Atlas (TCGA) and The Cancer Imaging Archive (TCIA), provide cancer genomic profiling and medical images counterpart, respectively [15,16], to promote cross-disciplinary research including radiogenomic studies [17,18,19].

In breast cancer, radiomic applications encompass diagnosis and better differentiation of malignant and benign entities as well as identifying ductal carcinoma in situ and invasive ductal carcinoma, for prognosis of metastatic potential, prediction of pathologic stage, lymph node involvement, molecular subtypes, and clinical outcomes [20,21,22,23,24,25,26,27,28,29,30].

Regarding the use of radiomic features for investigating breast cancer molecular receptor status, Agner et al. [28] extracted imaging features to differentiate TN cancers from other molecular subtypes. Yamaguchi et al. [29] investigated the relationship between heterogeneous kinetic curve pattern and molecular subtype. Blaschke et al. [31] showed that HER2-positive tumors have a greater uptake than other molecular subtypes. Li et al. [7] disclosed that the computer-aided tumor imaging phenotypes were able to differentiate between molecular prognostic biomarkers, and statistically significant associations between tumor phenotypes and receptor status were observed. Xie et al. [32] showed that whole-tumor MR multiparametric images offer a non-invasive analytical approach for breast cancer subtype classification and TN cancer cases. Guo et al. [21] demonstrated that the prediction performances by genomics alone, radiomics alone, and combined radiogenomic features showed statistically significant correlations with clinical outcomes (pathological receptors). Yoon et al. [33] used deep neural networks combining both radiomic and genomic features of invasive breast cancer, achieving high classification performance to predict pathological stage and molecular receptor status.

However, an important and often undervalued aspect in radiomic framework of analysis is features normalization relevance. In fact, data normalization methods are essential for radiomic features, due to their basic differences of scale, range, and statistical distributions. Untransformed features may have high levels of skewness, which can result in artificially low p-values in statistical analysis [34]. Moreover, neglecting feature normalization and the use of inappropriate normalization methods may lead to individual features being over or underrepresented and eventually introduce bias into developed models. Recently, normalization transformations are gaining huge interest in the era of machine learning (ML), especially in data preprocessing [35]. In the existing literature, standards to normalize quantitative radiomic features seem to be missing. None of the above-mentioned studies used a normalization method to decrease the overall bias that non-normalized features can generate in the analysis. Only Guo et al. [21] standardized the radiomic and genomic data before applying discriminative models. On the other side, several efforts have been shown to improve normalization procedures for MRI image intensity values as crucial preprocessing step. In fact, image variability and normalization steps are critical correction procedures for imaging-related batch effects before extracting quantitative radiomic features [36,37,38,39,40]. Although image preprocessing normalization steps are decisive to reduce technical variability across images, additional feature normalization steps are still needed and should be not overlooked. 

The aim of this study is to evaluate the impact of several normalization methods to study the relationship between radiomic features and breast tumor molecular receptor ER, PR, HER2, and TN status. We integrated data from TCIA-TCGA to analyze 36 MRI radiomic features extracted from 91 biopsy proven invasive breast cancers, T1-weighted Dynamic Contrast Enhancement (DCE) Magnetic Resonance Imaging (MRI), associated with tumor molecular receptor status.

Several feature normalization approaches such as scaling, z-score, robust z-score, log-transformation, quantile, upper quartile, and whitening methods were applied to radiomic features. We compared model predictive power and the impact of radiomic feature normalization using three advanced machine learning methods (Support Vector machine (SVM), Random Forest (RF) and Naïve Bayesian (NB) methods) to differentiate among ER, PR, HER2, and TN cases.

Finally, we included TCGA breast cancer miRNAs expression profiles to explore imaging-miRNA associations.

Our data highlighted that close attention is needed to assess image-based biomarker in radiomic analysis. Thus, the aim of this study is to provide several statistical approaches to generate quantitative MRI-based signature, which may lead to more precise breast cancer prognosis and help clinicians in decision-making towards personalized medicine.

## 2. Materials and Methods

### 2.1. Dataset

All patient data used in this study were obtained from The Cancer Genome Atlas (TCGA) (available at: https://tcga-data.nci.nih.gov, accessed October 9, 2019) Breast Cancer initiative. Patients were recruited from five comprehensive cancer centers across the United States. Imaging data processing and feature extraction were conducted by [7,21,27,41]. In order to enforce imaging uniformity, breast MRI studies were acquired by 1.5-Tesla (1.5 T) magnet strength using an MRI system from GE Medical Systems (Milwaukee, Wis). The final dataset included 91 breast MRI cases. 

#### 2.1.1. Clinical Data

Table 1 shows breast cancer histological type and molecular receptors status data (ER, PR, and HER2) carried out by Immunohistochemistry (IHC) test. The results of the IHC test can be 0 (negative), 1+ (also negative), 2+ (borderline), or 3+ (positive; the HER2 protein is overexpressed). In addition, TCGA also assessed HER2 receptor protein copies in the cancer cells by Fluorescence in Situ Hybridization (FISH). The ER and PR status for our dataset samples were obtained from the TCGA data portal [21]. The HER2 status of the samples was obtained from [15]. More clinical details are reported in Table S1. Ninety-one invasive breast carcinomas with radiomic imaging profiles were available from TCIA [15,21]. All samples were primary tumors from female patients. We investigated the prediction of ER, PR, and HER2 status and triple negative (TN) of patients using radiomic features alone.

#### 2.1.2. Image Data

All MRIs were acquired using a standard double-breast coil on a 1.5T GE whole-body MRI system (GE Medical Systems). In the current study, only T1-weighted, dynamic contrast-enhanced MR images were used. The imaging protocols included 1 pre-contrast image and 3 to 5 post-contrast images obtained using a T1-weighted, 3-dimensional (3D) spoiled gradient echo sequence with a gadolinium-based contrast agent (Omniscan; Nycomed-Amersham, Princeton, NJ). For further information refer to [7,21,27,41].

#### 2.1.3. Radiomic Features

A total of 36 MRI features, computer extracted image phenotypes (CEIPs), were calculated based on the automatically derived 3D tumor segmentations [7,21]. The CEIPs were divided into the following 6 phenotype categories: size (measuring tumor dimensions), shape (quantifying the 3D geometry), morphology (combining shape and margin characteristics), enhancement texture (describing the texture of the contrast uptake in the tumor on the first postcontrast MRIs), kinetic curve assessment (describing the shape of the kinetic curve and assessing the physiologic process of the uptake and washout of the contrast agent in the tumor during the dynamic imaging series), and enhancement-variance kinetics (characterizing the time course of the spatial variance of the enhancement within the tumor) [21]. Data were extracted using version V2010 of the UChicago [27]. The complete dataset was downloaded from the TCGA Breast Phenotype Research Group Data sets (available at: https://wiki.cancerimagingarchive.net/display/DOI/TCGA + Breast + Phenotype + Research + Group + Data + sets, accessed 9 October, 2019). Information about radiomic features, including feature names, label, description, and category is listed in Table S2 and as reported by [21].

#### 2.1.4. miRNA expression data

TCGA breast invasive carcinoma miRNA expression quantification data (raw read count, February 2018) produced on Illumina HiSeq 2000 sequencers (Illumina Ventures, San Diego, CA, USA) were downloaded using the free software R (R version 3.5.2) [42] and TCGAbiolinks R package [43].

TCGA-formatted miRNA-seq data according to the BCGSC miRNA Profiling Pipeline produces miRNA IDs with raw read counts of primary tumor (n = 1096 files for 1078 tumor cases) and normal solid tissue (n = 104 files for 104 normal cases). The miRNA raw counts (n = 1881 miRNAs), were processed as described in [44]. Then, we considered 1625 filtered and normalized miRNAs. We associated the molecular receptor ER, PR, HER2 status from [15], and filtering out NA receptor status. 

Samples derived from patients with (n = 504) and without (n = 57) breast cancer were then matched with the 91 patients included in the radiomic analysis. A total of 75 samples along with the respective radiomic and genomic features were included in the radiogenomic framework. 

### 2.2. Statistical Methods

Statistical analyses were carried out for radiomic features and genomic features alone to investigate the statistical significance of the features in detecting molecular receptor status. Associations between radiomics and genomics were also investigated in terms of correlation. 

Shapiro–Wilk test was used to determine the normality of radiomic and genomic features [45].

Wilcoxon signed-rank test and Fisher’s exact test were adopted to compare continuous and categorical clinical variables, respectively, between molecular receptor status (Table S1). R software (R Core Team. R: A language and environment for statistical computing. R Foundation for Statistical Computing, Vienna, Austria; http://www.R-project.org, 2019) was used to perform statistical analyses. 

#### 2.2.1. Radiomic Statistical Analysis

Seven different normalization techniques were used to normalize radiomic features. Features were standardized as the min–max normalization (i.e., scaling method), where each feature was normalized in the range from 0 to 1; z-score normalization, where each feature was normalized as *z* = (*x* − $\bar{x}$)/*s*, where *x*, $\bar{x}$, and *s* are the feature, the mean, and the standard deviation respectively [46]; robust z-score normalization is calculated from the median absolute deviation and median absolute deviation [47]; log-transformation (base 10), a constant value *a = b – min(x)* where *b* is 1 and *x* is the feature, was added to the data for handling negative values [48]; the upper quartile normalization divides each read count by the 75^th^ percentile of the read counts in its sample [49]; quantile normalization, which transform the original data to remove unwanted technical variation by forcing the observed distributions to be the same and the average distribution, obtained by taking the average of each quantile across samples, is used as the reference [50,51]; lastly, whitening normalization technique from the principle component analysis (PCA), is based on a linear transformation that converts a vector of random variables with a known covariance matrix into a set of new variables whose covariance is the identity matrix, meaning that they are uncorrelated and each have variance equal to one [52].

To investigate the effect of normalization techniques on the radiomic features, Spearman’s rank correlation was run between non-normalized radiomic features and normalized radiomic features for each of the seven different normalization methods. A Spearman’s *ρ* value greater than 0.8 and significant p-value (< 0.05) between non-normalized and normalized feature (using scaling, Z-score, robust Z-score, log-transformation, upper quartile, quantile, and whitening methods) was set as threshold to identify the normalized radiomic features that were in agreement with the non-normalized radiomic features. Bland–Altman analysis was also used to investigate the agreement between the features that showed a Spearman’s *ρ* value less than 0.08 and significant p-value. Bland–Altman procedure was used to compute 95% LoA (Limits of Agreement) [53].

For each binary problem, Wilcoxon signed-rank test was performed to investigate radiomic feature variation between two different groups (ER+ vs ER–, PR+ vs PR–, HER2+ vs HER2–, TN vs Others). Median trend or feature trend, using the following convention, was also investigated [54,55,56]:

- Two arrows, ↓↓ (or ↑↑) were used to report a significant (p-value < 0.05) decrease (or increase) of radiomic feature median in negative receptor status (ER–, PR–, HER2 +, TN);
- One arrow was used for non-significant variations: ↓ (or ↑) indicated a non-significant (p-value > 0.05) decrease (or increase) of a radiomic feature median in negative receptor status (ER–, PR–, HER2+, TN).

The statistical analysis was repeated for each normalization procedure described above. Non-parametric tests such as Wilcoxon signed-rank test and Spearman’s rank correlation were used since most of the radiomic features (>90%) were non-normally distributed in the Shapiro–Wilk test. A p-value less than 0.05 was considered significant. Holm’s correction was used for multiple hypothesis correction if necessary. 

**Figure 1:**
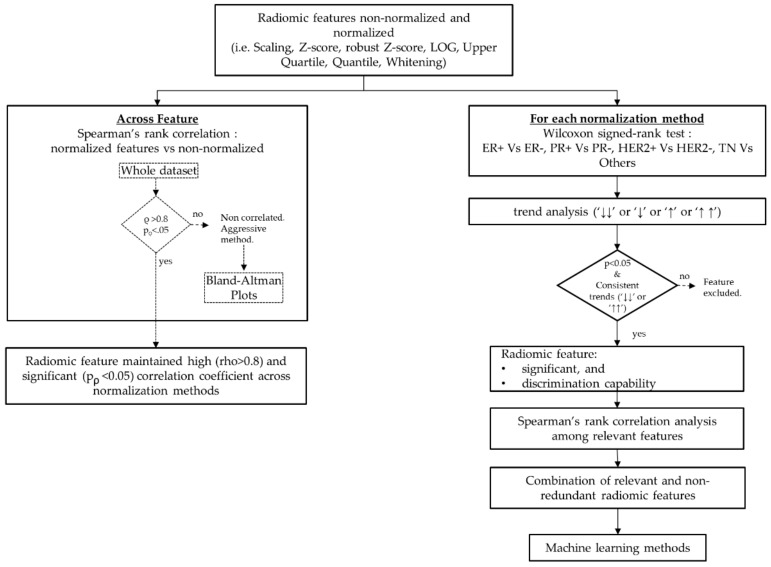
Radiomic analysis framework.

A synthetic scheme of the radiomic analysis carried out in this study is shown in Figure 1.

#### 2.2.2. Genomic Statistical Analysis

Starting from normalized counts we evaluated differentially expressed miRNAs between tumor and normal conditions, applying the Empirical Bayes method for differential expression analysis, edgeR R package [57], combined with the Generalized Linear Model approach (GLM). Differentially Expressed miRNAs (DEmiRNAs) with a |log2 fold-change |> 1.0 (hsa-mir-135b has LogFC 0.93) and with adjusted p-values (FDR) ≤ 0.05, were defined as significant and used for downstream analysis. In addition, differentially expressed miRNAs were analyzed through the use of Ingenuity Knowledge Base, IPA (QIAGEN Inc., https://www.qiagenbioinformatics.com/products/ingenuitypathway-analysis) to assess breast cancer implications.

We investigated DEmiRNAs expression as function of the ER, PR, HER2, and TN molecular receptor status and performed the Wilcoxon signed-rank test for statistical significance. 

A p-value less than 0.05 was considered significant. Holm’s correction was used for multiple hypothesis correction if necessary.

#### 2.2.3. Radiogenomic Statistical Analysis

After identifying the statistically significant radiomic features for each receptor, Spearman’s rank correlation was run between DEmiRNAs for each molecular receptor status (ER+ and ER–, PR+ and PR–, HER2+ and HER2–, TN and Others) and radiomic features. In the correlation analysis, two normalization methods were chosen for the radiomic features: upper quartile normalization method as both data types are normalized by upper quartile normalization method, and whitening-transformation method as it is the only normalization method that highlighted a significant change among radiomic features for all receptors’ status.

### 2.3. Machine Learning Classification 

The dataset was stratified random split per patient into two folders: Folder 1 (60%) was used to train and validate the classifiers; folder 2 (40%) to test the models. The reason of this split is that a classifier should be tested on an independent set of data to reduce overfitting problems and bias in the overall accuracy of the classifier [55,58]. 

#### 2.3.1. Feature Selection Methods

Feature selection is a critical step to build a robust model. In fact, the number of features used in the final classifier and its cardinality should be limited by the number of subjects presenting the event to detect in order to minimize the overfitting risk in a machine learning model. Moreover, a significant small set of clinical features powerfully simplifies the clinical interpretation of the results, by pointing the attention only on the most informative and relevant features [58]. Therefore, the feature selection process was based on two main steps: relevance analysis and redundancy analysis [55]. The former was performed using the Wilcoxon signed-rank test to identify the features that changed significantly between two conditions (binary problem). The latter selected only one feature from each cluster of features mutually correlated using Spearman’s rank correlation to reduce multicollinearity in the models [55]. 

#### 2.3.2. Training, Validation, and Testing 

Clinical outcomes have unbalanced ratio, which do not meet the assumptions of most machine learning-based models. To tackle this problem, Synthetic Minority Over-sampling Technique (SMOTE) was applied to balance the datasets [59]. It has been shown that SMOTE is a robust technique to overcome unbalanced dataset problems with a variety of classifiers [59,60]. In this study, SMOTE has shown to outperform other sampling methods and thus, it was used to balance the datasets.

Three different machine-learning approaches were considered to develop classifiers aiming to automatically classify receptor status based on MRI phenotypes: Support Vector Machine (SVM), which belongs to a general field of kernel-based machine learning methods and is used to classify both linearly and non-linearly separable data [61]; Random Forest decision trees, an ensemble learning method for classification that operates by constructing a multitude of decision trees during training and outputting the class that is the mode of the classes (classification) [62,63]; Bayesian classifier, a family of simple "probabilistic classifiers" based on applying Bayes’ theorem with strong (naïve) independence assumptions between the features [64].

Regarding model parameters, for SVM, polynomial kernel function with the degree from 1 to 5 was used. Random Forest decision trees were developed by changing confidence factor for pruning from 0.05 to 0.5 and minimum number of instances per leaf from 2 to 20. The algorithm parameters were tuned during training on folder 1. Each of those methods was used with all the combinations of relevant and non-redundant radiomic features. 

The training of the machine-learning models (including classifier parameter tuning) was performed on folder 1 (around 60% of the total number of patients). Folder 1 was also employed to validate the classifier using a k-fold cross-validation technique. The 3-fold person-independent cross-validation approach was used to validate the models in folder 1 [55]. The model was then tested on folder 2 (around 40% of the total number of patients), in order to assess their ability to automatically detect the receptor status. Binary classification performance measures were adopted according to standard formulae reported in Table 2 [65].

#### 2.3.3. Best Model Selection 

To evaluate the effect of normalization approaches for radiomic analysis, binary performance was calculated for each ML method across all normalization methods employed in this study. The best normalization technique for each ML model was selected as the one achieving the highest sensitivity, specificity, accuracy and area under the curve (AUC), and the classifier that employed a smaller number of radiomic features (i.e., less computational complexity model). Among the three different ML methods used to train, validate and test the classifiers (SVM, RF, BN), the best-performing model was chosen as the classifier achieving the highest AUC, which is a reliable estimator of both sensitivity and specificity rates; in case of equal AUC, the classifier with less computational complexity was chosen. 

## 3. Results

This study was performed on 91 invasive breast cancers with radiomic imaging profiles from female patients. The patients’ average age was 53.6 ± 11.5 years (range of 29–82 years). According to the clinical variables, no statistically significant differences were observed between receptor status (Table S1). For radiogenomic framework, we considered 75 matched samples of 91 invasive breast cancers.

### 3.1. Radiomics

**Figure 2:**
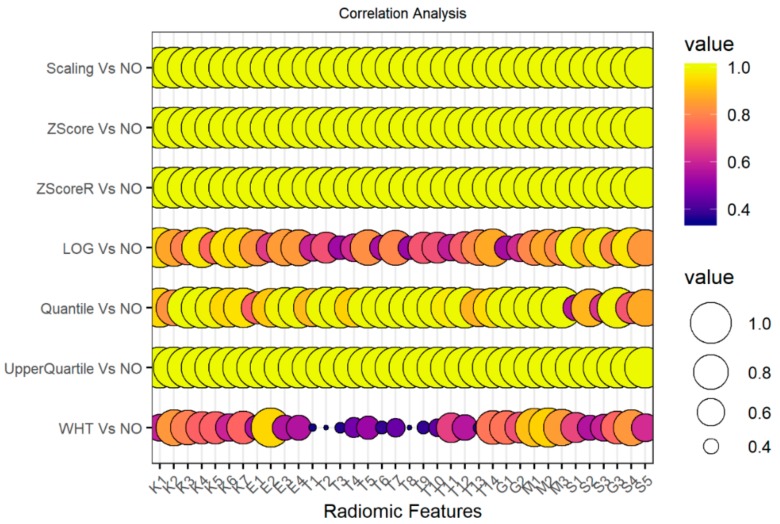
Correlation analysis on the whole dataset between non-normalized and normalized radiomic features. On the x-axis radiomic features are reported; on the y-axis correlation coefficients via Spearman correlation analysis are reported for each comparison between normalization methods and raw features (i.e., non-normalized radiomic features). All correlation p-values resulted less than 0.05. NO: non-normalized features; Scaling normalization method; Z-score normalization method; ZscoreR: Robust Z-score normalization method; LOG transformation; Quantile and Upper Quartile normalization method; WHT: Whitening normalization method.

A correlation analysis was run on the whole dataset to investigate the relationship between non-normalized and normalized radiomic features (i.e., Scaling, Z-Score, Robust Z-Score, Log transformation (LOG), Upper Quartile, Quantile and Whitening (WHT) methods). Spearman’s rank correlation was used. Table S3 showed ***ρ*** values of the radiomic features with a p-value < 0.05. A graphical representation of the correlation results is presented in Figure 2. 

As shown in Table S3 and Figure 2, non-normalized radiomic features are perfectly correlated with scaling, z-score, robust z-score and upper quartile normalized features. Between LOG transformation method and non-normalized features, only four radiomic features (E1, S1, S3, and S4) showed to be poorly correlated (*ρ* less than 0.8). Sixteen and thirty out of 36 radiomic features showed a very poor correlation value with the row radiomic features (i.e., non-normalized features) for the quantile and whitening normalization methods respectively.

Bland Altman analysis was also performed to visually investigate the agreement and disruption of normalized methods on the radiomic features that showed a Spearman’s rank coefficient less than 0.8 with non-normalized radiomic features. Therefore, the correlation analysis was supported by the visual inspection of the Bland–Altman analysis. An increase in bias and in width of the 95% limits of agreement (LoA) was observed for the radiomic features that showed a Spearman’s rank coefficient less than 0.8.

#### 3.1.1. Statistical Analysis per Receptor Status: ER+ vs ER–

Table 3 reports the results of the statistical analysis from Wilcoxon signed-rank test indicating association between MRI phenotype and receptor status ER+ versus ER–, for each normalization method. In Table 3 the radiomic features that showed a significant p-values were reported. 

**Figure 3:**
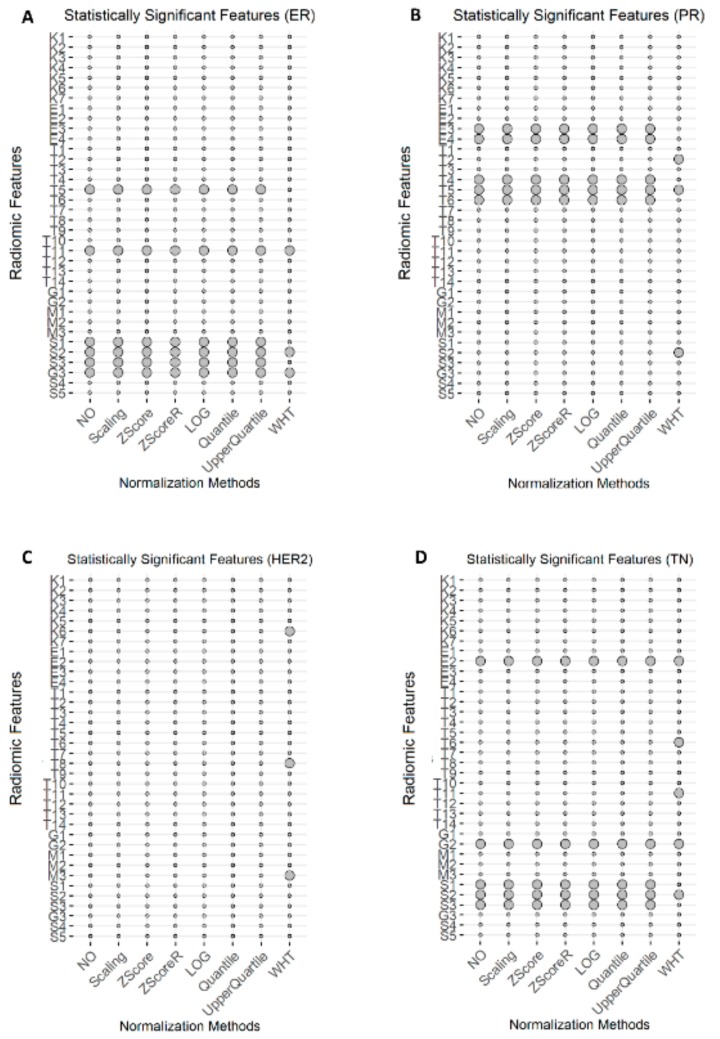
Statistically significant radiomic features. Statistically significant radiomic features across normalization methods are identified with a circle. (**A**) Statistically significant radiomic features for receptor status ER across normalization methods. (**B**) Statistically significant radiomic features for receptor status PR across normalization methods. (**C**) Statistically significant radiomic features for receptor status HER2 across normalization methods. (**D**) Statistically significant radiomic features for receptor status TN across normalization methods. NO: non-normalized features; Scaling normalization method; Z-score normalization method; ZscoreR: Robust Z-score normalization method; LOG transformation; Quantile and Upper Quartile normalization method; WHT: Whitening normalization method.

As shown in Table 3 and Figure 3A, 6 out of the 36 radiomic features (T5, T11, S1, S2, S3 and G3) showed significant changes between ER– and ER+ for non-normalized, scaling, z-score, robust z-score, log-transformation and upper quartile radiomic features. Three out of these 6 features (T5, T11, G3) decreased significantly in ER– cases, while the remaining 3 features showed a significant increase trend. For quantile normalization, five features out of 36 radiomic features (T5, T11, S2, S3, and G3) changed significantly between ER– and ER+. As far as whitening normalization method is concerned, only three radiomic features (T11, S2, and G3) showed significant changes between ER– and ER+ and maintained coherent trends with the other normalization methods. 

#### 3.1.2. Statistical Analysis per Receptor Status: PR+ vs PR–

Table 4 reports the results of the statistical analysis from Wilcoxon signed-rank test indicating association between MRI phenotype and receptor status PR+ versus PR–, for each normalization method. In Table 4 the radiomic features that showed a significant p-values were reported. 

As shown in Table 4 and Figure 3B, 5 out of the 36 radiomic features (E3, E4, T4, T5, and T6) showed significant changes between PR– and PR+ for non-normalized, scaling, z-score, robust z-score, log-transformation, upper quartile and quantile radiomic features. Three out of these 5 features (E3, E4, T5) showed a significantly decreased value in PR– status, while the remaining 2 features showed a significant increase. As far as whitening normalization method is concerned, only three radiomic features (T2, T5, and S2) showed significant changes between PR– and PR+. T2 and T5 showed a decreased value in PR– status, while S2 showed an increased value in PR– status. 

#### 3.1.3. Statistical Analysis per Receptor Status: HER2+ vs HER2–

Table 5 reports the results of the statistical analysis from Wilcoxon signed-rank test indicating association between MRI phenotype and receptor status HER2+ versus HER2–, for each normalization method. In Table 5 the radiomic features that showed a significant p-values were reported.

As shown in Table 5 and Figure 3C, none of 36 radiomic features showed significant changes between HER2– and HER2+ for all normalization methods but whitening transformation. As far as whitening normalization method is concerned, three radiomic features (K6, T8, and M3) showed significant changes between HER2– and HER2+. All these features showed an increased value in HER2 positive cases. 

#### 3.1.4. Statistical Analysis per Receptor Status: TN vs Others

Table 6 reports the results of the statistical analysis from Wilcoxon Rank test indicating association between MRI phenotype and receptor status TN versus Others, for each normalization method. In Table 6 the radiomic features that showed a significant p-values were reported. 

As shown in Table 6 and Figure 3D, 5 out of the 36 radiomic features (E2, G2, S1, S2, and S3) showed significant changes between TN vs Others for non-normalized, scaling, z-score, robust z-score, log-transformation, upper quartile, and quantile radiomic features. All 5 features showed a significantly increased value in TN status. As far as whitening normalization method is concerned, five different radiomic features (E2, T6, T11, G2, and S2) showed significant changes between TN and Others. All features, expect T11, showed an increased value in TN status. 

### 3.2. Genomics

As previously shown, several MRI radiomic features showed statistically significant variations in ER, PR, HER2, and TN status across different normalization approaches. 

Aberrant miRNAs expression is one of the genomic alterations occurring in breast cancer [66], which showed association with some MRI radiomic features [22] Thus, we computed miRNAs differentially expressed among TCGA breast tumor tissues with ER, PR, and HER2 receptor status annotation available. Ten differentially expressed miRNAs (DEmiRNAs, p adjusted ≤0.05), including hsa-mir-4662a, hsa-mir-486.1, hsa-mir-486.2, hsa-mir-526b(mir-515 family), hsa-mir-122, hsa-mir-653, hsa-mir-9.2, hsa-mir-135a.2, hsa-mir-184 and hsa-mir-206 (mir-1 family) were used to investigate relationship between radiomic and genomic features.

Box plots show DEmiRNAs trend as function of ER (Figure S1), PR (Figure S2), HER2 (Figure S3), and triple negative (Figure S4) receptor status, with Wilcoxon test for statistical significance (threshold p-value less than 0.05). Several DEmiRNAs showed to be statistically significant in ER receptor status (hsa-mir-122, hsa-mir-653, hsa-mir-9.2, hsa-mir-135a.2, hsa-mir-184) with a p-value < 0.05 (Figure S1). Three DEmiRNAs expression (hsa-mir-653, hsa-mir-9.2, hsa-mir-184) showed a significant p-values for PR status (Figure S2). Only two DEmiRNAs expression (hsa-mir-653, hsa-mir-135a.2) showed a significant variation in HER2 status (Figure S3). Lastly, four DEmiRNAs expression (hsa-mir-122, hsa-mir-653, hsa-mir-9.2, and hsa-mir-184) had a statistical variation in TN cases (Figure S4).

### 3.3. Radiogenomics 

As shown previously, a set of statistically significant radiomic features emerged from different normalization approaches. In order to assess imaging-genomic associations, considering that the distributions of radiomic and genomic features were not normal, we carried out Spearman’s correlation analysis between the statistically significant radiomic features and miRNAs differentially expressed in breast cancer.

Regarding the correlation, we chose radiomic features from two normalization methods: *i)* the upper quartile (UQ) normalized method, since the UQ method was used to normalize miRNA-seq data, and *ii)* the whitening method (WHT), because it provided statistically significant radiomic features for all receptors’ status.

The samples were stratified into ER+/ER–, PR+/PR–, and HER+/HER2– and TN status. Greater attention was paid in investigating imaging-genomic associations for ER-negative, PR-negative, HER2-positive, and TN cases, which are more relevant to the clinical practise. 

#### 3.3.1. Correlation Analysis per Receptor Status: ER+ vs ER–

**Figure 4:**
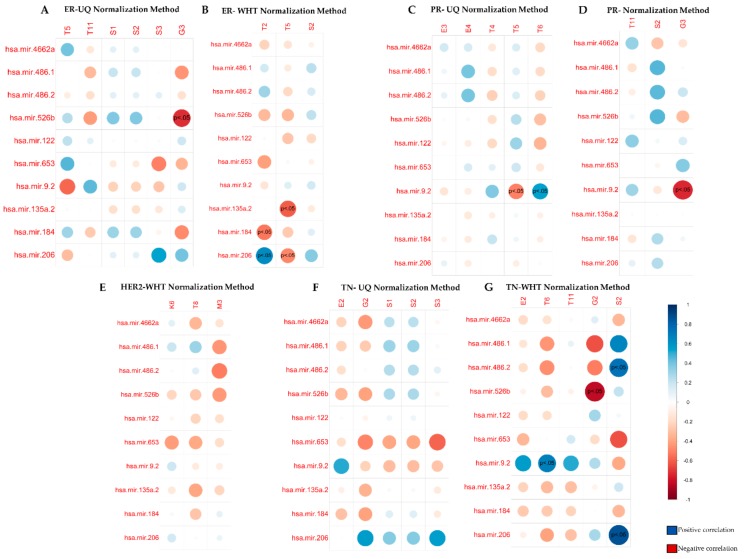
Spearman correlation between miRNAs expression and MRI radiomic features normalized by Upper Quartile and Whitening methods for molecular receptor status. (**A**) Correlation between ER negative breast cancer miRNAs expression and MRI radiomic features normalized by Upper Quartile (UQ) method. (**B**) Correlation between ER negative breast cancer miRNAs expression and MRI radiomic features normalized by Whitening (WHT) method. (**C**) Correlation between PR negative breast cancer miRNAs expression and MRI radiomic features normalized by Upper Quartile (UQ) method. (**D**) Correlation between PR negative breast cancer miRNAs expression and MRI radiomic features normalized by Whitening (WHT) method. (**E**) Correlation between HER2 positive breast cancer miRNAs expression and MRI radiomic features normalized by whitening methods. (**F**) Correlation between TN negative breast cancer miRNAs expression and MRI radiomic features normalized by Upper Quartile (UQ) method. (**G**) Correlation between TN negative breast cancer miRNAs expression and MRI radiomic features normalized by Whitening (WHT) method.

Spearman’s correlation analysis between UQ normalized radiomic features and miRNAs, for ER negative receptor status, highlighted a statistically significant negative correlation between the shape feature G3 and hsa-mir-526b (p-value adjusted < 0.05, Table S4), representing the ratio surface area to volume based on 3D reconstruction of lesion (Figure 4A). Other correlations included the size feature S3 with hsa-mir-653 and hsa-mir-206, characterizing lesion surface area, and the enhancement texture feature T5, indicating image homogeneity, associated with hsa-mir-9.2 (Figure 4A), although these correlations were not statistically significant (Table S4). 

In contrast, when we performed Spearman’s correlations for ER negative receptor status, using WHT normalized radiomic features, the shape feature G3 resulted associated with hsa-mir-9.2 (p adjusted < 0.01) in a statistically significant manner (Figure 4B, Table S4).

#### 3.3.2. Correlation Analysis per Receptor Status: PR+ vs PR–

For PR negative receptor status, UQ normalized radiomic features T5 (angular second moment, energy) and T6 (entropy) inversely correlated with hsa-mir-9.2 (p-value adjusted < 0.05) (Figure 4C, Table S4). These enhancement texture features indicate image homogeneity and randomness of the grey levels.

Similarly, for PR negative status, WHT normalized radiomic features (T5 and T2) showed to be correlated (p-value adjusted < 0.05) with different miRNAs (hsa-mir-135a.2, hsa-mir-184, hsa-mir-206) (Figure 4D, Table S4).

#### 3.3.3. Correlation Analysis per Receptor Status: HER2+ vs HER2–

For HER2 positive receptor status, we only performed a correlation analysis using WHT normalized radiomic features, as it was the only normalization method that identified statistically significant radiomic features. In this case, the morphological feature M3 correlated with hsa-mir-486.2, characterizing lesion enhancement structure from the central to a radial pattern, although this association was not statistically significant (Figure 4E, Table S4).

#### 3.3.4. Correlation Analysis per Receptor Status: TN vs Others 

We found interesting findings for TN receptor status, comparing UQ and WHT normalized radiomic features.

Correlation analysis of UQ normalized radiomic features highlighted that G2 and S3 were inversely correlated with hsa-mir-653 and hsa-mir-206, respectively (Figure 4F), whereas we found a positive correlation between the enhancement texture feature E2 and hsa-mir-9.2, indicating enhancement variance kinetics. However, none of these correlations were statistically significant (Table S4). 

Conversely, when we used WHT normalized radiomic features, we found several statistically significant correlations. Indeed, negative correlations were found between the shape feature G2 measuring irregularity of the lesion with hsa-mir-526b (p adjusted < 0.01), hsa-mir-486-1 and hsa-mir-486-2 (both, p-adjusted n.s.) (Figure 4G, Table S4).We found positive correlations with the size feature S2 indicating effective diameter of a sphere with the same volume of the lesion with hsa-mir-206 (p-value adjusted < 0.01), hsa-mir-486-1 (p-value adjusted n.s.) and hsa-mir-486-2 (p-adjusted < 0.05) and negative correlation with hsa-mir-653 (p-adjusted n.s.) (Figure 4G, Table S4). In addition, we found positive correlations between the enhancement variance kinetics E2, enhancement texture T6 and T11 features with hsa-mir-9.2. Among these associations, only T6 was statically significant (p-value adjusted < 0.05) (Figure 4G, Table S4).

### 3.4. Machine Learning Per Molecular Classification

Regarding the feature selection process, all possible combinations of relevant and non-redundant radiomic features were investigated for each normalization methods employed in the study. Each machine learning method was trained and validated with all combinations of radiomic features using folder 1. The best feature combination was chosen as the one achieving the best AUC during training. The classifiers were, then, tested on folder 2. 

#### 3.4.1. Receptor Status: ER– vs ER+ 

**Figure 5:**
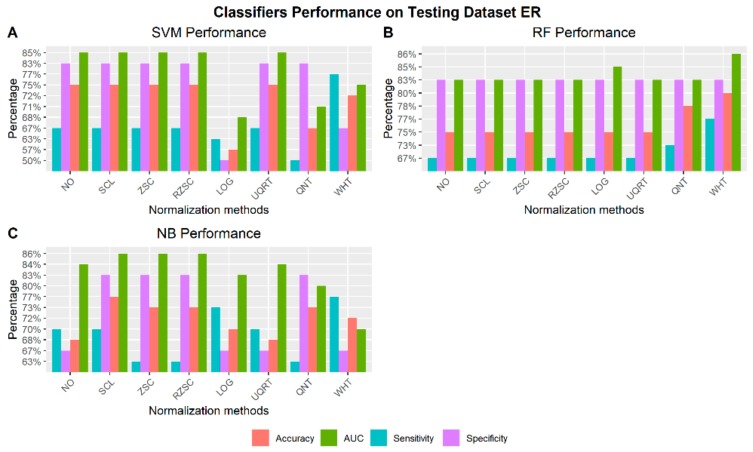
Classifiers performance on testing dataset to identify ER receptor status via radiomic features across normalization methods (NO: non-normalized features; SCL: Scaling normalization method; ZSC: Z-score normalization method; RZSC: Robust Z-score normalization method; LOG transformation; UPQRT: Upper Quartile normalization methods; QNT: Quantile normalization method; WHT: Whitening normalization method). **A**) Support Vector Machine (SVM) Performance on Testing dataset ER+ vs ER–. **B**) Random Forest (RF) Performance on Testing dataset ER+ vs ER–. **C**) Naïve Bayesian (NB) Performance on Testing dataset ER+ vs ER–.

Figure 5 shows the best performance of the three machine learning classifiers (SVM, RF, and NB) across all normalization methods to automatically detect ER receptor status. For each normalization approach the radiomic features chosen as the one achieving the best performance are reported along with the respective performance in Tables S5–S7.

**Figure 6:**
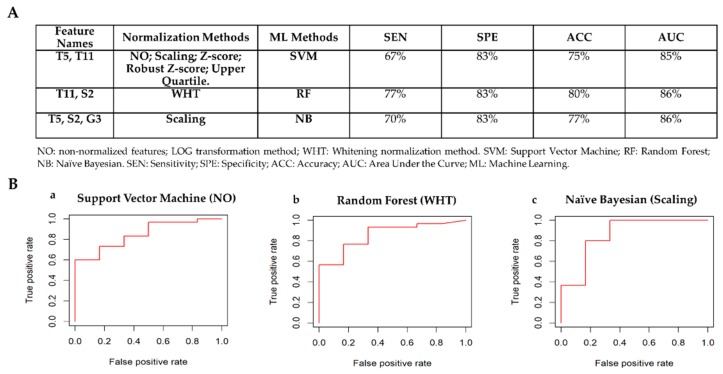
Classifiers’ performance to detect ER receptor status. (**A**) Comparison table among classifiers: ER+ vs ER–. (**B**) ROC curves for the best classifiers to automatically detect ER receptor status. a) Best classifier for Support Vector Machine Method. The normalization methods that achieved the best performance are Scaling, Z-score, Robust Z-score, Upper Quartile normalization methods. They achieved the same performance; therefore, one ROC curve with non-normalized features is reported. b) Best classifier for Random Forest Method. The normalization method that achieved the best performance is the whitening method. c) Best classifier for Naïve Bayesian Method. The normalization method that achieved the best performance is scaling method.

Figure 6A shows the best classifiers for each of the machine learning methods according to the criteria defined in Section 2.3.3. The classifier achieving the best performances to detect ER– status is Random Forest using only two radiomic features (T11 and S2) normalized via whitening method. Figure 6B shows the ROC curves for the best classifiers to automatically detect ER receptor status. Box plots of radiomic features chosen by the machine learning methods to automatically detect ER status are shown in Figure S5.

#### 3.4.2. Receptor Status: PR– vs PR+

**Figure 7:**
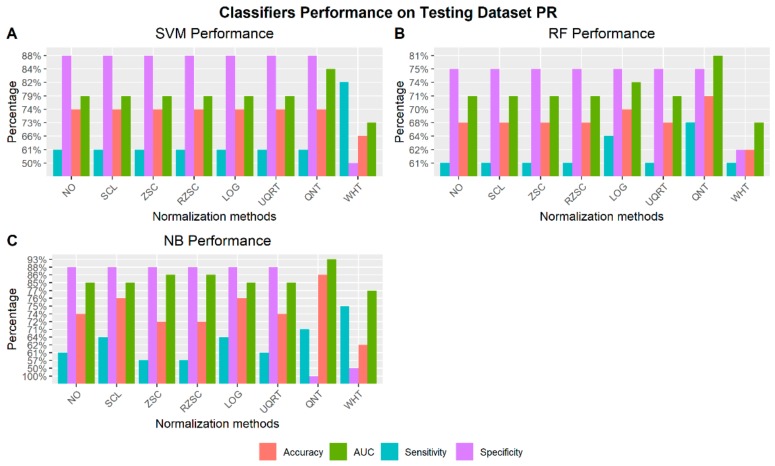
Classifiers performance on testing dataset to identify PR receptor status via radiomic features across normalization methods (NO: non-normalized features; SCL: Scaling normalization method; ZSC: Z-score normalization method; RZSC: Robust Z-score normalization method; LOG transformation; UPQRT: Upper Quartile normalization methods; QNT: Quantile normalization method; WHT: Whitening normalization method). A) Support Vector Machine (SVM) Performance on Testing dataset PR+ vs PR–. B) Random Forest (RF) Performance on Testing dataset PR+ vs PR–. C) Naïve Bayesian (NB) Performance on Testing dataset PR+ vs PR–.

Figure 7 shows the best performance of the three machine learning classifiers (SVM, RF, and NB) across all normalization methods to automatically detect PR receptor status. For each normalization approach the radiomic features chosen as the one achieving the best performance are reported along with the respective performance in Tables S8–S10.

**Figure 8:**
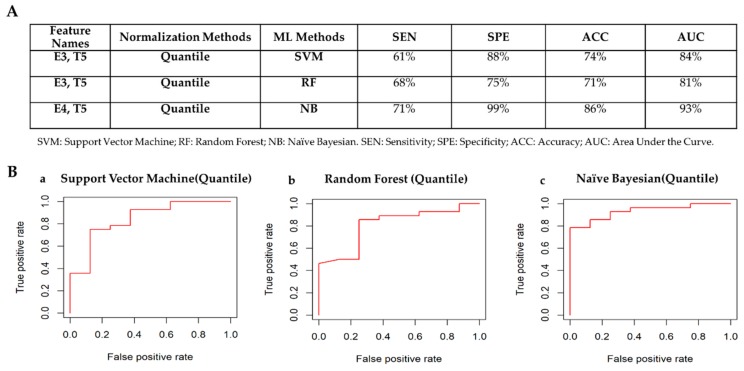
Classifiers’ performance to detect PR receptor status. (**A**) Comparison table among classifiers: PR+ vs PR–. (**B**) ROC curves for the best classifiers to automatically detect PR receptor status. a) Best classifier for Support Vector Machine Method. The normalization methods that achieved the best performance is the quantile normalization method. b) Best classifier for Random Forest Method. The normalization method that achieved the best performance is the quantile method. c) Best classifier for Naïve Bayesian Method. The normalization method that achieved the best performance is quantile method.

Figure 8A shows the best classifiers for each of the machine learning methods according to the criteria defined in Section 2.3.3. The classifier achieving the best performances to detect PR negative status is Naïve Bayesian model using only two radiomic features (E4 and T5) normalized via quantile method. Figure 8B shows the ROC curves for the best classifiers to automatically detect PR receptor status. Box plots of radiomic features chosen by the machine learning methods to automatically detect PR status are shown in Figure S6.

#### 3.4.3. Receptor Status: HER2– vs HER2+

**Figure 9:**
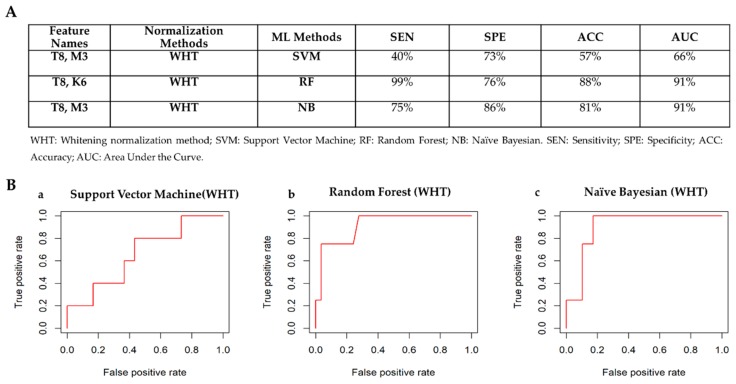
Classifiers’ performance to detect HER2 receptor status. (**A**) Comparison table among classifiers: HER2+ vs HER2–. (**B**) ROC curves for the best classifiers to automatically detect HER2 receptor status. Radiomic Feature normalized by whitening methods were considered for the classification task. a) Best classifier for Support Vector Machine Method. b) Best classifier for Random Forest Method. c) Best classifier for Naïve Bayesian method.

Figure 9A shows the best classifiers for each of the machine learning methods according to the criteria defined in Section 2.3.3. The classifier achieving the best performances to detect HER2 receptor status is Random Forest model using only two radiomic features (T8 and K6) normalized via whitening normalization method. Figure 9B shows the ROC curves for the best classifiers to automatically detect HER2 receptor status. Box plots of radiomic features chosen by the machine learning methods to automatically detect HER2 status are shown in Figure S7.

#### 3.4.4. Receptor Status: TN vs Others

**Figure 10:**
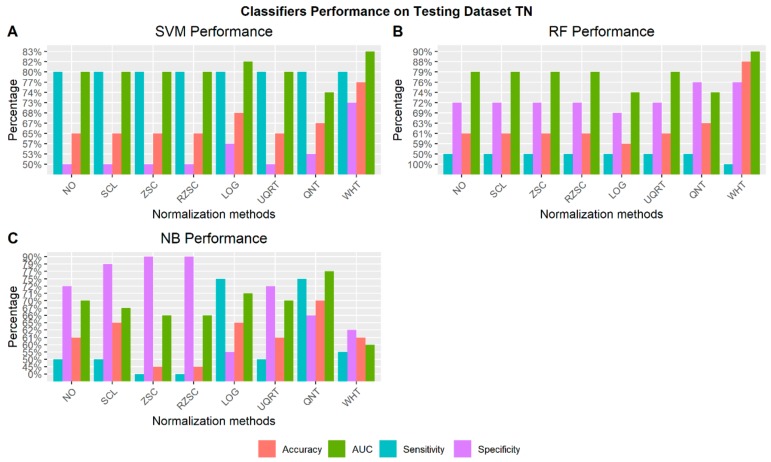
Classifiers performance on testing dataset to identify TN cases via radiomic features across normalization methods (NO: non-normalized features; SCL: Scaling normalization method; ZSC: Z-score normalization method; RZSC: Robust Z-score normalization method; LOG transformation; UPQRT: Upper Quartile normalization methods; QNT: Quantile normalization method; WHT: Whitening normalization method). **A**) Support Vector Machine (SVM) Performance on Testing dataset TN vs Others. **B**) Random Forest (RF) Performance on Testing dataset TN vs Others. **C**) Naïve Bayesian (NB) Performance on Testing dataset TN vs Others.

Figure 10 shows the best performance of the three machine learning classifiers (SVM, RF, and NB) across all normalization methods to automatically detect TN cases. For each normalization approach the radiomic features chosen as the one achieving the best performance are reported along with the respective performance in Tables S11–S13. 

**Figure 11:**
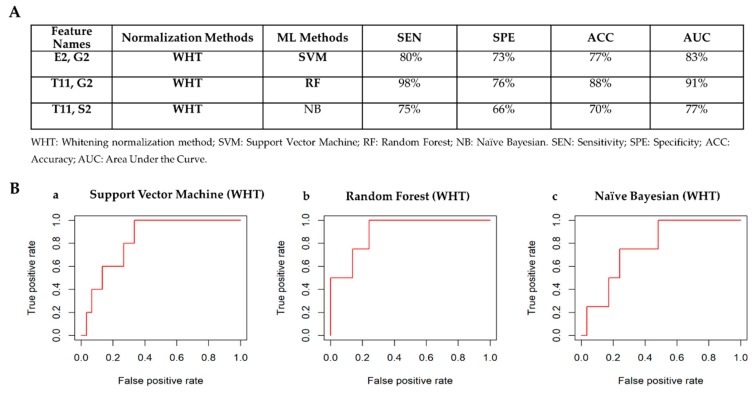
Classifiers’ performance to detect TN receptor status. (**A**) Comparison table among classifiers: TN vs Others. (**B**) ROC curves for the best classifiers to automatically detect TN receptor status. a) Best classifier for Support Vector Machine Method. The normalization methods that achieved the best performance is the quantile normalization method. b) Best classifier for Random Forest Method. The normalization method that achieved the best performance is the quantile method. c) Best classifier for Naïve Bayesian Method. The normalization method that achieved the best performance is quantile method.

Figure 11A shows the best classifiers for each of the machine learning methods according to the criteria defined in Section 2.3.3. The classifier achieving the best performances to detect TN cases is Random Forest model using only two radiomic features (T11 and G2) normalized via whitening method. Figure 11B shows the ROC curves for the best classifiers to automatically detect TN cases. Box plots of radiomic features chosen by the machine learning methods to automatically detect TN cases are shown in Figure S8. 

## 4. Discussion

The results from this study demonstrate that quantitative radiomic analysis shows potential as a means for high-throughput image-based phenotyping to automatically detect breast cancer receptor status via machine learning methods. Moreover, this study investigates the effect of normalization methods on radiomic features to automatically detect receptor status of breast cancer patients. Altogether our results suggest that quantitative radiomic analysis is influenced by normalization method choice. 

The results from the correlation analysis along with visual inspection by Bland–Altman analysis, showed that all radiomic features are perfectly correlated with non-normalized radiomic features using scaling, z-score, robust z-score, and upper quartile normalization methods. Therefore, these methods help reducing bias and do not alter the information carried by the “row” radiomic features. These normalization methods, thus, result less aggressive than log-transformation, quantile, and whitening methods. In fact, four, sixteen and thirty out of 36 radiomic features showed a poorly correlation value with non-normalized radiomic features for log-transformation, quantile, and whitening normalization methods respectively. These results were expected. Log-transformation should be carefully applied to radiomic data as does not always decrease the skewness of the distribution but can make it more skewed than the raw data. Another common use of the log-transformation is to reduce the variability of data, especially in data sets including outliers, but it can often increase the variability of data. Moreover, log-transformation can only be used for positive outcomes, thus, for negative values it is common to add a small positive constant, *a*, to all observations before applying this transformation. Although this practice can appear quite meaningless, it can have a noticeable effect on the level of statistical significance [67]. Therefore, log-transformation should be carefully used in radiomic studies. 

Quantile normalization method transforms original data to remove undesirable technical variation. Technical variation could cause apparent differences between samples. This kind of normalization is achieved by forcing the observed distributions to be the same and the average distribution, obtained by taking the average of each quantile across samples, is used as the reference. This method has worked well in practice, but important information could be wiped out and features that are not statically different across samples can be artificially induced [51]. Therefore, although it could improve the predictive power of the features, it should be used carefully. 

Whitening normalization using PCA can make a more substantial normalization of the features to give it zero mean and unit covariance, so that transformed features become decorrelated [68]. One weakness of this transformation is that it can greatly exaggerate the noise in the data, since it stretches all dimensions (including noise) to be of equal size in the input. However, this method is often used as pre-processing step for machine learning models as it improves the overall performance. 

Consequently, “aggressive” normalization methods should be carefully assessed based on the nature of the data and the main goal of the analysis.

Regarding the statistical analysis per receptor status, the results are in agreement with the existing literature [69]. 

Our results indicate that ER-negative cases may have larger volume, diameter and lesion area whereas less homogeneity and brightness than ER-positive cases. Similar observations were also reported by Chen et al. [70] in a correlation study between ER status and breast MRI radiomics and Li et al. [7] that showed an higher effective diameter, irregularity and entropy in ER-negative cases. 

Different results were observed using quantile and whitening transformation methods. In fact, less features showed to be significantly different between positive and negative ER cases. One features (S1) and three features (T5, S1, S3) were not statistically significant using whitening and quantile transformation methods compared to the other investigated normalization methods. Moreover, these phenotypes captured on imaging are in agreement with prior literature showing that ER– tumors have higher microvessel density [71], higher levels of vascular endothelial growth factor [72], and higher proliferative activity [72].

Our results indicate that PR-negative cases may have lower enhancement-variance rate and homogeneity while higher entropy. PR-negative cases are also larger, more irregular in shape, more heterogeneous, and have a faster contrast uptake than PR-positive cases as also demonstrated by Li et al. [7]. These observations confirm the existing evidence that PR cancers tend to have high growth factor signaling [73]. Different results were observed using whitening-transformation method. In fact, less features showed to be significantly different between positive and negative PR cases. Three radiomic features showed significant changes between PR cases, while T5 was also captured as significant feature (p-value < 0.05) by other normalization methods, T2 and S2 were not statistically significant using other transformation methods.

As far as HER2 growth factor is concerned, none of 36 radiomic features showed significant changes between positive and negative HER2 cases except for whitening-transformation. Indeed, features normalized by whitening method showed an increased value in enhancement texture, structure and voxel for HER2 positive case. 

TN cases showed to be larger, more irregular in shape, heterogeneous, and have a faster contrast uptake rate than all the other cases. Similar results were achieved by Agner et al. [28] showing more lesion heterogeneity in TN breast cancers than non-TN cancers. Li et al. [7] reported that TN cases are larger in size due to the over expression of oncogenes that favor cell proliferation defined by the absence of ER, PR, and HER2 receptors. Youk et al. [74] showed that larger tumor size was significantly associated with TN breast cancer. Using whitening transformation, different radiomic features showed statistical changes between cases but maintained the overall trends. 

Overall, imaging phenotypes like enhancement texture, size features convey pathophysiologic characteristics like proliferation and angiogenesis provide clues to more accurate prognosis and optimal treatment. It is interesting to note that enhancement texture (especially heterogeneity) emerged as an important discriminatory indicator. In our study, a decrease in homogeneity was observed in ER–, PR–, and TN cancers relative to ER+, PR+, and non-TN cancers, in agreement with the exiting literature. Enhancement texture homogeneity was also found to be positively associated with tumor stage, suggesting that larger, heterogeneous tumors are potentially linked to more aggressive cancers [7] with higher probability of recurrence [41].

Moreover, we questioned whether radiomic features may be associated with aberrantly expressed breast cancer miRNAs. Thus, we evaluated the correlation degree using two different radiomic features normalization methods. In accordance with the exiting literature, breast miRNAs expression resulted associated especially to MR radiomic features such as shape and enhancement texture features [22]. However, as expected, we showed that various radiomic features normalization approaches capture different imaging-genomic associations. For example, for ER receptor status the upper quartile normalized G3 feature correlated with hsa-mir-526b whereas whitening normalized G3 feature correlated with hsa-mir-9.2. For this reason, once chosen the appropriate radiomic framework of analysis, conspicuous testing of radiomic results on external and independent image datasets are encouraged.

Using machine learning algorithms, enhancement texture features were selected as the most predictive feature for tumor receptor status. Hence, radiomic features have a high predictive power to detect clinical variables related to the genomic status of a tumor, such as ER status and PR status. The machine learning method that better performed to automatically detect ER, HER2, and TN cases is the Random Forest with an AUC of 86%, 91%, and 91% respectively. Naïve Bayesian outperformed the other methods to detect PR cases with 93% AUC. 

Compared to the existing literature, the results achieved in this study via machine learning methods outperformed other studies. 

Xie et al. [32] achieved an AUC of 91% via linear SVM using several whole-tumor texture features extracted from DCE and DWI-related original images, while we achieved the same AUC values using only two DCE imaging feature, reducing considerably the computational complexity of the Random Forest model. Agner et al. [28] used the morphologic and texture features extracted from the whole tumor on the early postcontrast images in conjunction with an SVM classifier, achieving a lower AUC (of 74%) to detect TN cases. Li at al. [7] also achieved a lower AUC value using quantitative imaging phenotypes of size, shape, and enhancement texture via linear discriminant analysis to predict clinical receptor status, namely ER+ vs ER– (89% AUC), PR+ vs PR– (69% AUC), HER2+ vs HER2– (65%AUC),and TN cases (67% AUC). Yoon et al. [33] used a deep learning approach utilizing imaging data from TCIA to generate prediction of clinical outcomes receptor status. They achieved lower performance to predict ER (82% AUC), PR (75% AUC), HER2 (72% AUC), but they accomplished a slightly higher AUC values using the combination of radiomics and genomics data. 

Overall, in this study using robust machine learning methods and employing few radiomic feature to reduce the computational complexity of the ML models, we achieved better AUC values to discriminate clinical receptor status, paving the way to non-invasive disease monitoring using imaging features as potential surrogate markers of underlying molecular activity that may aid in clinical diagnosis and treatment planning.

Most of the radiomic features chosen by the ML methods were normalized using whitening transformation, whereas to detect PR cases the radiomic features were normalized by quantile transformation. These results were expected as quantile and whitening transformation are considered among the best normalizing transformation when compared to other alternatives in machine learning models [52,75]. Both transformation methods reduce the leverage of potentially influential points among candidate predictors and remove redundancy among them. In fact, normalization of the covariates mitigates the leverage and potential influence of these covariates to an extent, which in some cases, will allow for more robust model selection. 

However, this study presents some limitations. The MR images used in this study were acquired by different institutions more than 10 years ago. These may have had different acquisition protocols, different weight-based dosing regimen for contrast agents, and different time resolution of post-contrast sequences, and therefore, these images may not reflect current MRI technology, which has advanced considerably during the past decade. Consequently, our results can only be generalized to this population. However, the bias was limited by performing several normalization methods on the extracted radiomic features. Another limitation of our study was that the patient sample was relatively small and unbalanced, but by performing a cross validation for each molecular classification assessment (e.g., between ER+ and ER– cases) and applying balancing methods (SMOTE) to the developed model, the overall bias was reduced. In addition, our study lacks further validation on internal cohort, for which patient recruitment is ongoing. 

Therefore, after this pilot study, which helped us establishing a robust framework of analysis, upcoming work will include studies on a larger and more recent clinically annotated data set to verify and validate the results from this preliminary study. We will further assess the role of the MRI phenotypes in combination with genomic and clinical information to improve the prediction power of the machine learning-based models. Furthermore, molecular breast cancer intrinsic subtypes will be also investigated.

## 5. Conclusions

In this study, our results demonstrate that there are statistically significant associations between radiomic tumor features and breast cancer molecular receptor status. Breast tumor characterization, including the ER, PR, and HER2 molecular receptor status, represents a primary goal to direct treatment options and targeted chemotherapies. Thus, in order to achieve a more accurate and early diagnosis is relevant to identify non-invasive diagnostic imaging biomarker associated to the molecular receptor status. This study investigated several statistical approaches to identify the relationship between medical imaging quantitative descriptors of clinical phenotypes and molecular tumor characteristics. Moreover, the prediction power of each radiomic feature normalization method was assessed by three advanced machine learning algorithms. The results from this study offer a better understanding into the underlying tumor biology including tumor enhancement texture. Identifying receptor status via imaging phenotypes may aid in clinical diagnosis and treatment planning. At the same time, we aware researchers to implement in radiomic analysis framework different radiomic feature normalization approaches and standards for post-acquisition data processing, in order to ensure more robust findings. Another important advantage of our approach is that opens the door to the identification of non-invasive in vivo imaging surrogate markers that could reflect the underlying tumor biology. 

## Figures and Tables

**Table 1:** Clinical Data.

Variables	Number
*Breast Cancer Types*	
Ductal carcinoma	79
Lobular carcinoma	10
Mixed	2
*Molecular Receptor Status*	
*Estrogen Receptor (ER)*	
Positive	76
Negative	15
*Progesterone Receptor (PR* *)*	
Positive	71
Negative	20
*Human Epidermal Growth Factor Receptor 2 (HER2)*	
Positive	12
Negative	74
*Triple Negative (TN)*	
Triple negative (ER–, PR–, HER2–)	12
Others	74

**Table 2:** Binary performance measures.

Measure	Formula
Total Classification Accuracy (ACC)	$\left(TP+TN\right)/\left(TP+TN+FP+FN\right)$
Sensitivity (SEN)	$TP/\left(TP+FN\right)$
Specificity (SPE)	$TN/\left(FP+TN\right)$
Area Under the Curve (AUC)	-

True positive (TP); true negative (TN), false negative (FN); false positive (FP).

**Table 3:** Results from the Wilcoxon signed-rank test indicating association between MRI phenotype and molecular classification ER+ versus ER–.

	Non-Normalized	Scaling	Z-Score	Robust Z-Score	LOG	Quantile	Upper Quartile	WHT
Feature Names	Pval (Trend)	Pval (Trend)	Pval (Trend)	Pval (Trend)	Pval (Trend)	Pval (Trend)	Pval (Trend)	Pval (Trend)
T5	0.029 (↓↓)	0.029 (↓↓)	0.029 (↓↓)	0.029 (↓↓)	0.029 (↓↓)	0.029 (↓↓)	0.029 (↓↓)	0.287 (↓)
T11	0.031 (↓↓)	0.031 (↓↓)	0.031 (↓↓)	0.031 (↓↓)	0.031 (↓↓)	0.031 (↓↓)	0.031 (↓↓)	0.002 (↓↓)
S1	0.003 (↑↑)	0.003 (↑↑)	0.003 (↑↑)	0.003 (↑↑)	0.003 (↑↑)	0.003 (↑↑)	0.003 (↑↑)	0.688 (↑)
S2	0.003 (↑↑)	0.003 (↑↑)	0.003 (↑↑)	0.003 (↑↑)	0.003 (↑↑)	0.003 (↑↑)	0.003 (↑↑)	0.004 (↑↑)
S3	0.011 (↑↑)	0.011 (↑↑)	0.011 (↑↑)	0.011 (↑↑)	0.011 (↑↑)	0.011 (↑↑)	0.011 (↑↑)	0.524 (↓)
G3	0.029 (↓↓)	0.029 (↓↓)	0.029 (↓↓)	0.029 (↓↓)	0.029 (↓↓)	0.029 (↓↓)	0.029 (↓↓)	0.029 (↓↓)

↓↓ (or ↑↑): used to report a significant (p-value < 0.05) decrease (or increase) of feature median in negative receptor status (ER–),↓ (or ↑) indicated a non-significant (p-value > 0.05) decrease (or increase) of a feature median in negative receptor status (ER–).

**Table 4:** Results from the Wilcoxon signed-rank test indicating association between MRI phenotype and molecular classification PR+ versus PR.

	Non-Normalized	Scaling	Z-Score	Robust Z-Score	LOG	Quantile	Upper Quartile	WHT
Feature Names	Pval (Trend)	Pval (Trend)	Pval (Trend)	Pval (Trend)	Pval (Trend)	Pval (Trend)	Pval (Trend)	Pval (Trend)
E3	0.021 (↓↓)	0.021 (↓↓)	0.021 (↓↓)	0.021 (↓↓)	0.021 (↓↓)	0.021 (↓↓)	0.021 (↓↓)	0.418 (↑)
E4	0.038 (↓↓)	0.038 (↓↓)	0.038 (↓↓)	0.038 (↓↓)	0.038 (↓↓)	0.038 (↓↓)	0.038 (↓↓)	0.867 (↑)
T2	0.071 (↓)	0.071 (↓)	0.071 (↓)	0.071 (↓)	0.071 (↓)	0.071 (↓)	0.071 (↓)	0.046 (↓↓)
T4	0.049 (↑↑)	0.049 (↑↑)	0.049 (↑↑)	0.049 (↑↑)	0.049 (↑↑)	0.049 (↑↑)	0.049 (↑↑)	0.178 (↑)
T5	0.004 (↓↓)	0.004 (↓↓)	0.004 (↓↓)	0.004 (↓↓)	0.004 (↓↓)	0.004 (↓↓)	0.004 (↓↓)	0.02 (↓↓)
T6	0.02 (↑↑)	0.02 (↑↑)	0.02 (↑↑)	0.02 (↑↑)	0.02 (↑↑)	0.02 (↑↑)	0.02 (↑↑)	0.303 (↑)
S2	0.175 (↑)	0.175 (↑)	0.175 (↑)	0.175 (↑)	0.175 (↑)	0.175 (↑)	0.175 (↑)	0.021 (↑↑)

↓↓ (or ↑↑): used to report a significant (p-value < 0.05) decrease (or increase) of feature median in negative receptor status (PR–), ↓ (or ↑) indicated a non-significant (p-value > 0.05) decrease (or increase) of a feature median in negative receptor status (PR–).

**Table 5:** Results from the Wilcoxon signed-rank test indicating association between MRI phenotype and molecular classification HER2+ versus HER2–.

	Non-Normalized	Scaling	Z-Score	Robust Z-Score	LOG	Quantile	Upper Quartile	WHT
Feature Names	Pval (Trend)	Pval (Trend)	Pval (Trend)	Pval (Trend)	Pval (Trend)	Pval (Trend)	Pval (Trend)	Pval (Trend)
K6	0.054 (↑)	0.054 (↑)	0.054 (↑)	0.054 (↑)	0.054 (↑)	0.054 (↑)	0.054 (↑)	0.008 (↑↑)
T8	0.414 (↑)	0.414 (↑)	0.414 (↑)	0.414 (↑)	0.414 (↑)	0.414 (↑)	0.414 (↓)	0.025 (↑↑)
M3	0.393 (↑)	0.393 (↑)	0.393 (↑)	0.393 (↑)	0.393 (↑)	0.393 (↑)	0.393 (↑)	0.048 (↑↑)

↓↓ (or ↑↑): used to report a significant (p-value<0.05) decrease (or increase) of feature median in negative receptor status (HER2+), ↓ (or ↑) indicated a non-significant (p-value > 0.05) decrease (or increase) of a feature median in negative receptor status (HER2+).

**Table 6:** Results from the Wilcoxon signed-rank test indicating association between MRI phenotype and molecular classification TN versus Others.

	Non- Normalized	Scaling	Z-Score	Robust Z-Score	LOG	Quantile	Upper Quartile	WHT
Feature Names	Pval (Trend)	Pval (Trend)	Pval (Trend)	Pval (Trend)	Pval (Trend)	Pval (Trend)	Pval (Trend)	Pval (Trend)
E2	0.048 (↑↑)	0.048 (↑↑)	0.048 (↑↑)	0.048 (↑↑)	0.048 (↑↑)	0.048 (↑↑)	0.048 (↑↑)	0.035 (↑↑)
T6	0.107 (↑)	0.107 (↑)	0.107 (↑)	0.107 (↑)	0.107 (↑)	0.107 (↑)	0.107 (↑)	0.024 (↑↑)
T11	0.078 (↓)	0.078 (↓)	0.078 (↓)	0.078 (↓)	0.078 (↓)	0.078 (↓)	0.078 (↓)	0.024 (↓↓)
G2	0.048 (↑↑)	0.048 (↑↑)	0.048 (↑↑)	0.048 (↑↑)	0.048 (↑↑)	0.048 (↑↑)	0.048 (↑↑)	0.019 (↑↑)
S1	0.01 (↑↑)	0.01 (↑↑)	0.01 (↑↑)	0.01 (↑↑)	0.01 (↑↑)	0.01 (↑↑)	0.01 (↑↑)	0.458 (↑)
S2	0.01 (↑↑)	0.01 (↑↑)	0.01 (↑↑)	0.01 (↑↑)	0.01 (↑↑)	0.01 (↑↑)	0.01 (↑↑)	0.021 (↑↑)
S3	0.012 (↑↑)	0.012 (↑↑)	0.012 (↑↑)	0.012 (↑↑)	0.012 (↑↑)	0.012 (↑↑)	0.012 (↑↑)	0.259 (↑)

↓↓ (or ↑↑): used to report a significant (p-value < 0.05) decrease (or increase) of feature median in negative receptor status (TN), ↓ (or ↑) indicated a non-significant (p-value > 0.05) decrease (or increase) of a feature median in negative receptor status (TN).

## Supplementary Materials

The following are available online at https://www.mdpi.com/2072-6694/12/2/518/s1, Figure S1: Relationship between breast cancer miRNAs expression and ER receptor status; Figure S2: Relationship between breast cancer miRNAs expression and PR receptor status; Figure S3: Relationship between breast cancer miRNAs expression and HER2 receptor status; Figure S4: Relationship between breast cancer miRNAs expression and TN cases; Figure S5. Box plots of radiomic features chosen by the machine learning methods to automatically detect ER status; Figure S6: Box plots of radiomic features chosen by the machine learning methods to automatically detect PR status; Figure S7: Box plots of radiomic features chosen by the machine learning methods to automatically detect HER2 receptor; Figure S8: Box plots of radiomic features chosen by the machine learning methods to automatically detect TN cases. Table S1: Additional Clinical Parameters; Table S2: Radiomic Features; Table S3: Correlation Analysis on the Whole Dataset between Non-Normalized and Normalized Radiomic Features; Table S4: Imaging-genomic associations; Table S5: Support Vector Machine Performance on Testing dataset ER+ vs ER–; Table S6: Random Forest Performance on Testing dataset ER+ vs ER–; Table S7: Naïve Bayesian Performance on Testing dataset ER+ vs ER–; Table S8: Support Vector Machine Performance on Testing dataset PR+ vs PR–; Table S9: Random Forest Performance on Testing dataset PR+ vs PR–: Table S10: Naïve Bayesian Performance on Testing dataset PR+ vs PR–; Table S11: Support Vector Machine Performance on Testing dataset TN vs Others; Table S12: Random Forest Performance on Testing dataset TN vs Others; Table S13: Naïve Bayesian Performance on Testing dataset TN vs Others.
