# Supplementary material for: The Impact of Normalization Approaches to Automatically Detect Radiogenomic Phenotypes Characterizing Breast Cancer Receptors Status
Source: Cancers (Basel). 2020 Feb 24;12(2):518. doi: 10.3390/cancers12020518 (PMC7072389; doi:10.3390/cancers12020518)
Supplement: Supplementary file 1 [file cancers-12-00518-s001.pdf]

Supplementary Materials

Table S1. Additional Clinical Parameters

	ER			PR			HER2			TN cases		
**Clinical Data**	**Negative**	**Positive**	**p-value**	**Negative**	**Positive**	**p-value**	**Negative**	**Positive**	**p-value**	**Others**	**TN**	**p-value**
**Age#, mean (SD)**	52.4 (13.18)	54.14 (11.45)	0.63	52.75 (12.87)	54.16 (11.41)	0.65	54.43(11.61)	49.83(13.20)	0,27	54.22 (11.86)	51.08 (12.11)	0.41
**Cancer metastasis stage code, n (%)**			0.53			0.45			0.56			0.56
cM0 (i+)	0 (0.0)	2 (2.6)		0 (0.0)	2 (2.8)		2 (2.7)	0 (0.0)		2 (2.7)	0 (0.0)	
M0	15 (100.0)	74 (97.4)		20 (100.0)	69 (97.2)		72 (97.3)	12 (100.0)		72 (97.3)	12 (100.0)	
**Neoplasm disease lymph node stage, n (%)**			0.15			0.42			0.17			0.11
N0	8 (53.3)	25 (32.9)		9 (45.0)	24 (33.8)		28 (37.8)	2 (16.7)		23 (31.1)	7 (58.3)	
N0 (i-)	2 (13.3)	9 (11.8)		3 (15.0)	8 (11.3)		11 (14.9)	0 (0.0)		9 (12.2)	2 (16.7)	
N0 (i+)	0 (0.0)	2 (2.6)		0 (0.0)	2 (2.8)		2 (2.7)	0 (0.0)		2 (2.7)	0 (0.0)	
N1	0 (0.0)	7 (9.2)		1 (5.0)	6 (8.5)		4 (5.4)	3 (25.0)		7 (9.5)	0 (0.0)	
N1a	2 (13.3)	20 (26.3)		4 (20.0)	18 (25.4)		16 (21.6)	5 (41.7)		20 (27.0)	1 (8.3)	
N1mi	0 (0.0)	5 (6.6)		0 (0.0)	5 (7.0)		3 (4.1)	1 (8.3)		4 (5.4)	0 (0.0)	
N2	1 (6.7)	0 (0.0)		1 (5.0)	0 (0.0)		1 (1.4)	0 (0.0)		0 (0.0)	1 (8.3)	
N2a	0 (0.0)	5 (6.6)		0 (0.0)	5 (7.0)		5 (6.8)	0 (0.0)		5 (6.8)	0 (0.0)	
N3	1 (6.7)	1 (1.3)		1 (5.0)	1 (1.4)		1 (1.4)	1 (8.3)		2 (2.7)	0 (0.0)	
N3a	1 (6.7)	1 (1.3)		1 (5.0)	1 (1.4)		2 (2.7)	0 (0.0)		1 (1.4)	1 (8.3)	
NX	0 (0.0)	1 (1.3)		0 (0.0)	1 (1.4)		1 (1.4)	0 (0.0)		1 (1.4)	0 (0.0)	
**Neoplasm disease stage, n (%)**			0.40			0.37			0.37			0.37
Stage I	3 (20.0)	14 (18.4)		3 (15.0)	14 (19.7)		3 (15.0)	14 (19.7)		3 (15.0)	14 (19.7)	
Stage IA	0 (0.0)	5 (6.6)		0 (0.0)	5 (7.0)		0 (0.0)	5 (7.0)		0 (0.0)	5 (7.0)	
Stage II	0 (0.0)	1 (1.3)		0 (0.0)	1 (1.4)		0 (0.0)	1 (1.4)		0 (0.0)	1 (1.4)	
Stage IIA	8 (53.3)	32 (42.1)		12 (60.0)	28 (39.4)		12 (60.0)	28 (39.4)		12 (60.0)	28 (39.4)	
Stage IIB	1 (6.7)	16 (21.1)		2 (10.0)	15 (21.1)		2 (10.0)	15 (21.1)		2 (10.0)	15 (21.1)	
Stage IIIA	1 (6.7)	6 (7.9)		1 (5.0)	6 (8.5)		1 (5.0)	6 (8.5)		1 (5.0)	6 (8.5)	
Stage IIIC	2 (13.3)	2 (2.6)		2 (10.0)	2 (2.8)		2 (10.0)	2 (2.8)		2 (10.0)	2 (2.8)	
**Breast cancer histologic type, n (%)**			0.81			0.60			0.52			0.92
Ductal	14 (93.3)	64 (84.2)		19 (95.0)	59 (83.1)		62 (83.8)	12 (100.0)		63 (85.1)	11 (91.7)	
Lobular	1 (6.7)	10 (13.2)		1 (5.0)	10 (14.1)		10 (13.5)	0 (0.0)		9 (12.2)	1 (8.3)	
Mixed	0 (0.0)	2 (0.8)		0 (0.0)	2 (2.4)		2 (2.8)	0 (0.0)		2 (2.8)	0 (0.0)	
**Lymph node count, n (%)**			0.54			0.49			0.16			0.45
0	0 (0.0)	1 (1.3)		0 (0.0)	1 (1.4)		1 (1.4)	0 (0.0)		1 (1.4)	0 (0.0)	
1	2 (13.3)	4 (5.3)		2 (10.0)	4 (5.7)		6 (8.2)	0 (0.0)		4 (5.5)	2 (16.7)	
2	1 (6.7)	12 (16.0)		1 (5.0)	12 (17.1)		12 (16.4)	0 (0.0)		11 (15.1)	1 (8.3)	
3	1 (6.7)	9 (12.0)		3 (15.0)	7 (10.0)		8 (11.0)	0 (0.0)		7 (9.6)	1 (8.3)	
4	1 (6.7)	7 (9.3)		1 (5.0)	7 (10.0)		6 (8.2)	1 (8.3)		6 (8.2)	1 (8.3)	
5	2 (13.3)	5 (6.7)		1 (5.0)	6 (8.6)		5 (6.8)	2 (16.7)		6 (8.2)	1 (8.3)	
6	1 (6.7)	3 (4.0)		2 (10.0)	2 (2.9)		3 (4.1)	1 (8.3)		3 (4.1)	1 (8.3)	
7	0 (0.0)	2 (2.7)		0 (0.0)	2 (2.9)		2 (2.7)	0 (0.0)		2 (2.7)	0 (0.0)	
8	0 (0.0)	1 (1.3)		0 (0.0)	1 (1.4)		0 (0.0)	1 (8.3)		1 (1.4)	0 (0.0)	
9	0 (0.0)	2 (2.7)		0 (0.0)	2 (2.9)		1 (1.4)	1 (8.3)		2 (2.7)	0 (0.0)	
10	0 (0.0)	1 (1.3)		1 (5.0)	0 (0.0)		1 (1.4)	0 (0.0)		1 (1.4)	0 (0.0)	
11	0 (0.0)	2 (2.7)		0 (0.0)	2 (2.9)		2 (2.7)	0 (0.0)		2 (2.7)	0 (0.0)	
12	0 (0.0)	3 (4.0)		1 (5.0)	2 (2.9)		2 (2.7)	1 (8.3)		3 (4.1)	0 (0.0)	
13	0 (0.0)	2 (2.7)		0 (0.0)	2 (2.9)		2 (2.7)	0 (0.0)		2 (2.7)	0 (0.0)	
14	0 (0.0)	1 (1.3)		0 (0.0)	1 (1.4)		1 (1.4)	0 (0.0)		1 (1.4)	0 (0.0)	
15	0 (0.0)	1 (1.3)		0 (0.0)	1 (1.4)		1 (1.4)	0 (0.0)		1 (1.4)	0 (0.0)	
16	1 (6.7)	1 (1.3)		1 (5.0)	1 (1.4)		1 (1.4)	1 (8.3)		2 (2.7)	0 (0.0)	
17	1 (6.7)	2 (2.7)		1 (5.0)	2 (2.9)		3 (4.1)	0 (0.0)		2 (2.7)	1 (8.3)	
18	0 (0.0)	3 (4.0)		0 (0.0)	3 (4.3)		2 (2.7)	0 (0.0)		2 (2.7)	0 (0.0)	
19	1 (6.7)	1 (1.3)		1 (5.0)	1 (1.4)		2 (2.7)	0 (0.0)		1 (1.4)	1 (8.3)	
20	1 (6.7)	0 (0.0)		1 (5.0)	0 (0.0)		1 (1.4)	0 (0.0)		0 (0.0)	1 (8.3)	
21	1 (6.7)	0 (0.0)		1 (5.0)	0 (0.0)		1 (1.4)	0 (0.0)		0 (0.0)	1 (8.3)	
23	0 (0.0)	1 (1.3)		0 (0.0)	1 (1.4)		0 (0.0)	1 (8.3)		1 (1.4)	0 (0.0)	
24	1 (6.7)	4 (5.3)		2 (10.0)	3 (4.3)		3 (4.1)	2 (16.7)		5 (6.8)	0 (0.0)	
26	0 (0.0)	2 (2.7)		0 (0.0)	2 (2.9)		2 (2.7)	0 (0.0)		2 (2.7)	0 (0.0)	
27	0 (0.0)	1 (1.3)		0 (0.0)	1 (1.4)		1 (1.4)	0 (0.0)		1 (1.4)	0 (0.0)	
28	0 (0.0)	2 (2.7)		0 (0.0)	2 (2.9)		2 (2.7)	0 (0.0)		2 (2.7)	0 (0.0)	
29	1 (6.7)	0 (0.0)		1 (5.0)	0 (0.0)		1 (1.4)	0 (0.0)		0 (0.0)	1 (8.3)	
32	0 (0.0)	1 (1.3)		0 (0.0)	1 (1.4)		1 (1.4)	0 (0.0)		1 (1.4)	0 (0.0)	
35	0 (0.0)	1 (1.3)		0 (0.0)	1 (1.4)		0 (0.0)	1 (8.3)		1 (1.4)	0 (0.0)	
**Margin status, n (%)**			0.35			0.26			0.41			0.41
Negative	15 (100.0)	68 (94.4)		20 (100.0)	63 (94.0)		69 (94.5)	12 (100.0)		69 (94.5)	12 (100.0)	
Positive	0 (0.0)	4 (5.6)		0 (0.0)	4 (6.0)		4 (5.5)	0 (0.0)		4 (5.5)	0 (0.0)	
**Menopause status, n (%)**			1.00			0.92			0.55			0.90
Peri	1 (6.7)	5 (6.8)		1 (5.0)	5 (7.4)		6 (8.5)	0 (0.0)		5 (7.0)	1 (8.3)	
Post	8 (53.3)	41 (56.2)		12 (60.0)	37 (54.4)		40 (56.3)	6 (50.0)		40 (56.3)	6 (50.0)	
Pre	6 (40.0)	27 (37.0)		7 (35.0)	26 (38.2)		25 (35.2)	6 (50.0)		26 (36.6)	5 (41.7)	
**Number of positive lymph nodes, n (%)**			0.11			0.15			0.05			0.20
0	10 (66.7)	38 (50.7)		13 (65.0)	35 (50.0)		43 (58.9)	2 (16.7)		36 (49.3)	9 (75.0)	
1	1 (6.7)	16 (21.3)		1 (5.0)	16 (22.9)		10 (13.7)	6 (50.0)		16 (21.9)	0 (0.0)	
2	1 (6.7)	7 (9.3)		2 (10.0)	6 (8.6)		5 (6.8)	2 (16.7)		6 (8.2)	1 (8.3)	
3	0 (0.0)	6 (8.0)		1 (5.0)	5 (7.1)		5 (6.8)	1 (8.3)		6 (8.2)	0 (0.0)	
4	0 (0.0)	3 (4.0)		0 (0.0)	3 (4.3)		3 (4.1)	0 (0.0)		3 (4.1)	0 (0.0)	
5	0 (0.0)	1 (1.3)		0 (0.0)	1 (1.4)		1 (1.4)	0 (0.0)		1 (1.4)	0 (0.0)	

6	0 (0.0)	1 (1.3)		0 (0.0)	1 (1.4)		1 (1.4)	0 (0.0)		1 (1.4)	0 (0.0)	
7	1 (6.7)	0 (0.0)		1 (5.0)	0 (0.0)		1 (1.4)	0 (0.0)		0 (0.0)	1 (8.3)	
9	0 (0.0)	1 (1.3)		0 (0.0)	1 (1.4)		1 (1.4)	0 (0.0)		1 (1.4)	0 (0.0)	
10	2 (13.3)	0 (0.0)		2 (10.0)	0 (0.0)		1 (1.4)	1 (8.3)		1 (1.4)	1 (8.3)	
13	0 (0.0)	1 (1.3)		0 (0.0)	1 (1.4)		1 (1.4)	0 (0.0)		1 (1.4)	0 (0.0)	
15	0 (0.0)	1 (1.3)		0 (0.0)	1 (1.4)		1 (1.4)	0 (0.0)		1 (1.4)	0 (0.0)	
**Prior diagnosis, n (%)**			0.30			0.05			1.00			0.26
No	14 (93.3)	75 (98.7)		18 (90.0)	71 (100.0)		72 (97.3)	12 (100.0)		73 (98.6)	11 (91.7)	
Yes	1 (6.7)	1 (1.3)		2 (10.0)	0 (0.0)		2 (2.7)	0 (0.0)		1 (1.4)	1 (8.3)	
**Race, n (%)**			0.52			0.21			1.00			0.46
Black	1 (6.7)	3 (4.0)		2 (10.0)	2 (2.9)		4 (5.4)	0 (0.0)		3 (4.1)	1 (8.3)	
White	14 (93.3)	72 (96.0)		18 (90.0)	68 (97.1)		70 (94.6)	11 (100.0)		70 (95.9)	11 (91.7)	

#continuous variable.

Table S2. Radiomic Features

Feature category	Label	Description
**Size Feature**	**S1**	Lesion volume (mm^3).
	**S2**	Effective diameter (mm).
	**S3**	Surface area (mm^2).
	**S4**	Maximum linear size (mm).
**Shape Feature**	**G1**	Sphericity. Similarity of the lesion shape to a sphere.
	**G2**	Irregularity. Deviation of the lesion surface from the surface of a sphere.
	**G3**	Surface-to-volume ratio (1⁄mm). Ratio of surface area to volume.
**Morphological features**	**M1**	Mean of the image gradient at the lesion margin.
	**M2**	Variance of the image gradient at the lesion margin.
	**M3**	Indicates how well the enhancement structure in a lesion extends in a radial pattern originating from the center of the lesion.
**Enhancement textures**	**T1**	Contrast. Measure of local image variations.
	**T2**	Correlation. Measure of image linearity.
	**T3**	Difference entropy. Measure of the randomness of the difference of neighboring voxels' gray levels.
	**T4**	Difference variance. Measure of variations of difference of gray levels between voxel pairs.
	**T5**	Angular second moment (energy). Measure of image homogeneity.
	**T6**	Entropy. Measure of the randomness of the gray levels.
	**T7**	Inverse difference moment. Measure of the image homogeneity.
	**T8**	Information measure of correlation 1. Measure of nonlinear gray-level dependence.
	**T9**	Information measure of correlation 2. Measure of nonlinear gray-level dependence.
	**T10**	Maximum correlation coefficient. Measure of nonlinear gray-level dependence.
	**T11**	Sum average. Measure of the overall image brightness
	**T12**	Sum entropy. Measure of the randomness of the sum of gray levels of neighboring voxels.
	**T13**	Sum variance. Measure of the spread in the sum of the gray levels of voxel-pairs distribution.
	**T14**	Sum of squares (variance). Measure of the spread in the gray-level distribution.
**Kinetic curve assessment**	**K1**	Maximum enhancement. Maximum contrast enhancement.
	**K2**	Time to peak (s). Time at which the maximum enhancement occurs.
	**K3**	Uptake rate (1⁄s). Uptake speed of the contrast enhancement.
	**K4**	Washout rate (1⁄s). Washout speed of the contrast enhancement.
	**K5**	Curve shape index. Difference between late and early enhancement.
	**K6**	Enhancement at first postcontrast time point. Enhancement at first postcontrast time point.
	**K7**	Signal enhancement ratio of initial enhancement to overall enhancement.
**Enhancement-variation kinetics**	**E1**	Maximum variance of enhancement Maximum spatial variance of contrast enhancement over time.
	**E2**	Time to peak at maximum variance (s). Time at which the maximum variance occurs.
	**E3**	Enhancement variance increasing rate (1⁄s). Rate of increase of the enhancement variance during uptake.
	**E4**	Enhancement variance decreasing rate (1⁄s). Rate of decrease of the enhancement variance during washout.

Table S3. Correlation Analysis on the Whole Dataset between Non-Normalized and Normalized Radiomic Features.

	**Scaling Vs NO**	**Z-Score Vs NO**	**Robust Z-Score Vs NO**	**LOG Vs NO**	**Upper Quartile Vs NO**	**Quantile Vs NO**	**Whitening Vs NO**
**Feature Label**	ρ	ρ	ρ	ρ	ρ	ρ	ρ
**E1**	1	1	1	**0.726**	1	0.835	**0.549**
**E2**	1	1	1	0.921	1	**0.647**	0.944
**E3**	1	1	1	0.991	1	0.846	**0.555**
**E4**	1	1	1	0.999	1	0.841	**0.555**
**G1**	1	1	1	0.998	1	**0.537**	**0.767**
**G2**	1	1	1	0.999	1	**0.619**	**0.694**
**G3**	1	1	1	0.991	1	0.803	**0.743**
**K1**	1	1	1	0.945	1	0.969	**0.592**
**K2**	1	1	1	0.831	1	0.866	0.839
**K3**	1	1	1	1	1	**0.796**	**0.791**
**K4**	1	1	1	1	1	0.971	**0.731**
**K5**	1	1	1	0.993	1	**0.743**	**0.731**
**K6**	1	1	1	0.946	1	0.959	**0.601**
**K7**	1	1	1	0.961	1	0.952	**0.738**
**M1**	1	1	1	0.999	1	0.807	0.907
**M2**	1	1	1	1	1	0.868	0.935
**M3**	1	1	1	1	1	0.811	0.848
**S1**	1	1	1	**0.571**	1	0.995	**0.671**
**S2**	1	1	1	0.893	1	0.894	**0.556**
**S3**	1	1	1	**0.635**	1	0.987	**0.595**
**S4**	1	1	1	**0.707**	1	0.968	0.836
**S5**	1	1	1	0.872	1	0.839	**0.617**
**T1**	1	1	1	0.901	1	**0.596**	**0.351**
**T2**	1	1	1	0.999	1	**0.696**	**0.347**
**T3**	1	1	1	0.997	1	**0.526**	**0.364**
**T4**	1	1	1	0.934	1	**0.618**	**0.465**
**T5**	1	1	1	1	1	0.823	**0.521**
**T6**	1	1	1	0.998	1	**0.542**	**0.381**
**T7**	1	1	1	1	1	0.804	**0.447**
**T8**	1	1	1	1	1	**0.519**	**0.347**
**T9**	1	1	1	1	1	**0.705**	**0.379**
**T10**	1	1	1	0.999	1	**0.692**	**0.435**
**T11**	1	1	1	0.974	1	**0.581**	**0.659**
**T12**	1	1	1	1	1	**0.716**	**0.562**
**T13**	1	1	1	0.889	1	0.815	**0.371**
**T14**	1	1	1	0.951	1	0.871	**0.771**

In bold the radiomic features that showed a Spearman's rank coefficient (ρ) less than 0.8. All radiomic features showed a significant p-value. NO: Non-normalized radiomic features.

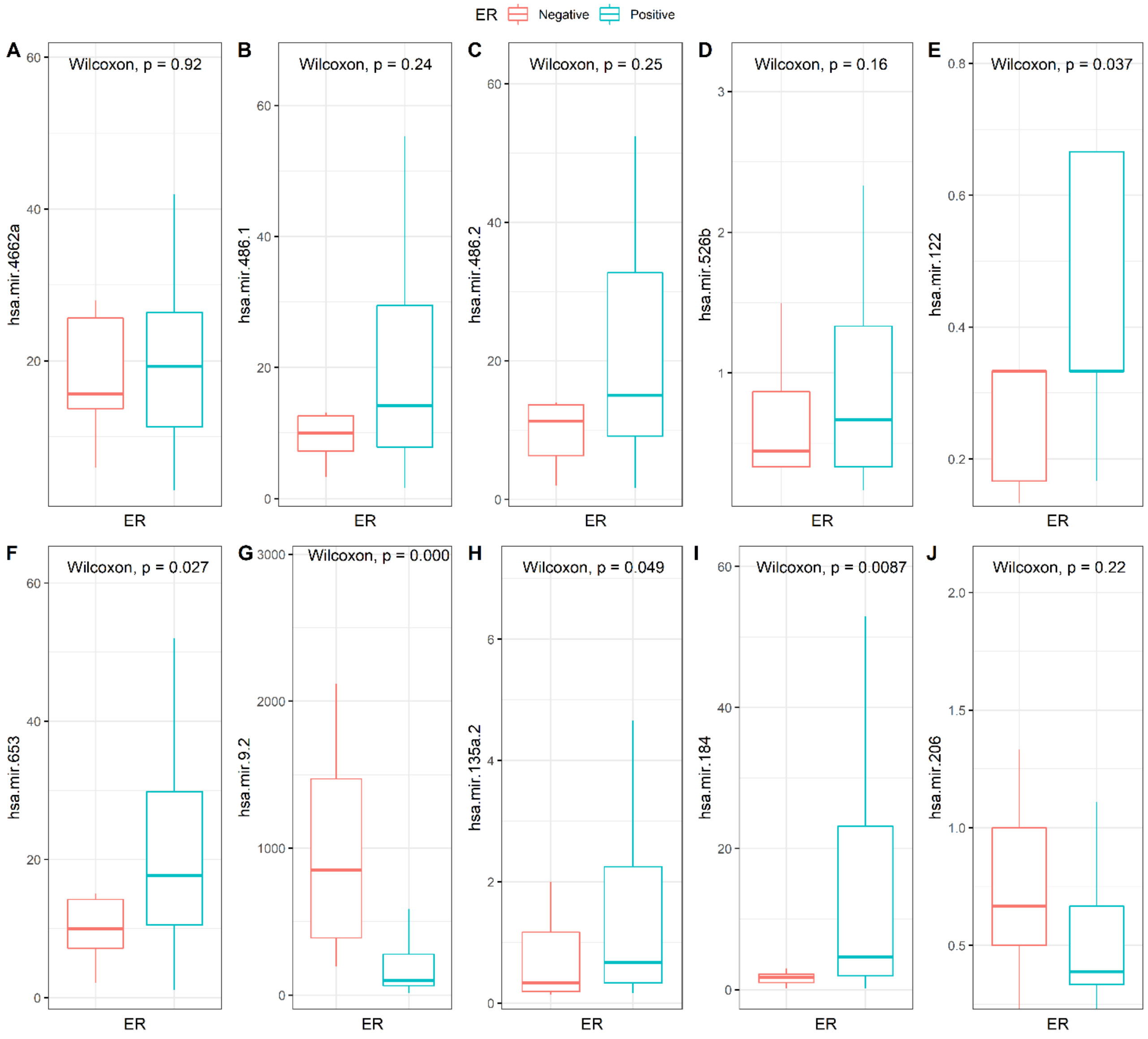

Figure S1. Relationship between breast cancer miRNAs expression and ER receptor status. (A-J) Extracted breast cancer miRNAs expression with associated p-values calculated using Wilcoxon sign rank test.

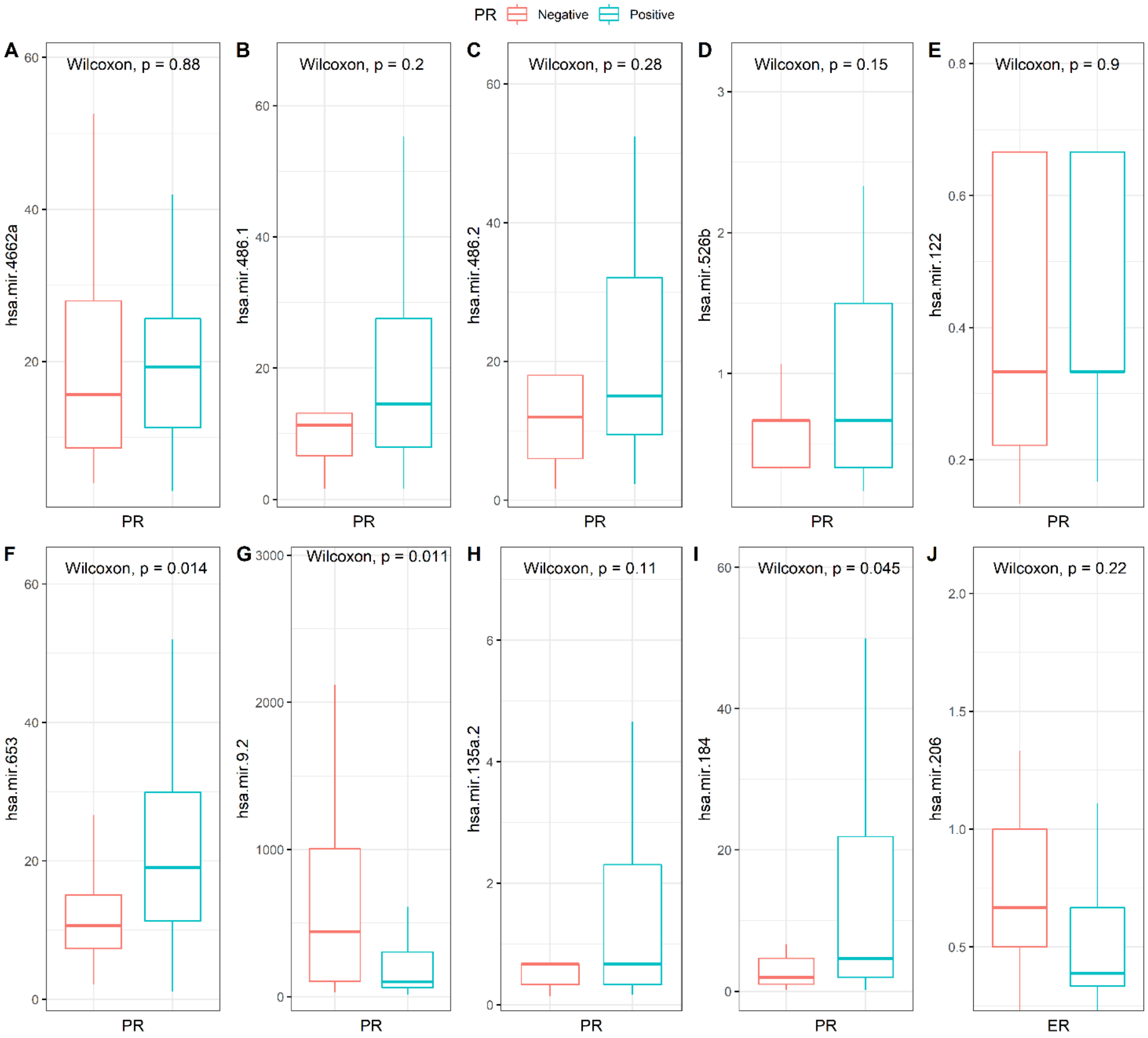

Figure S2. Relationship between breast cancer miRNAs expression and PR receptor status. (A-J) Extracted breast cancer miRNAs expression with associated p-values calculated using Wilcoxon sign rank test.

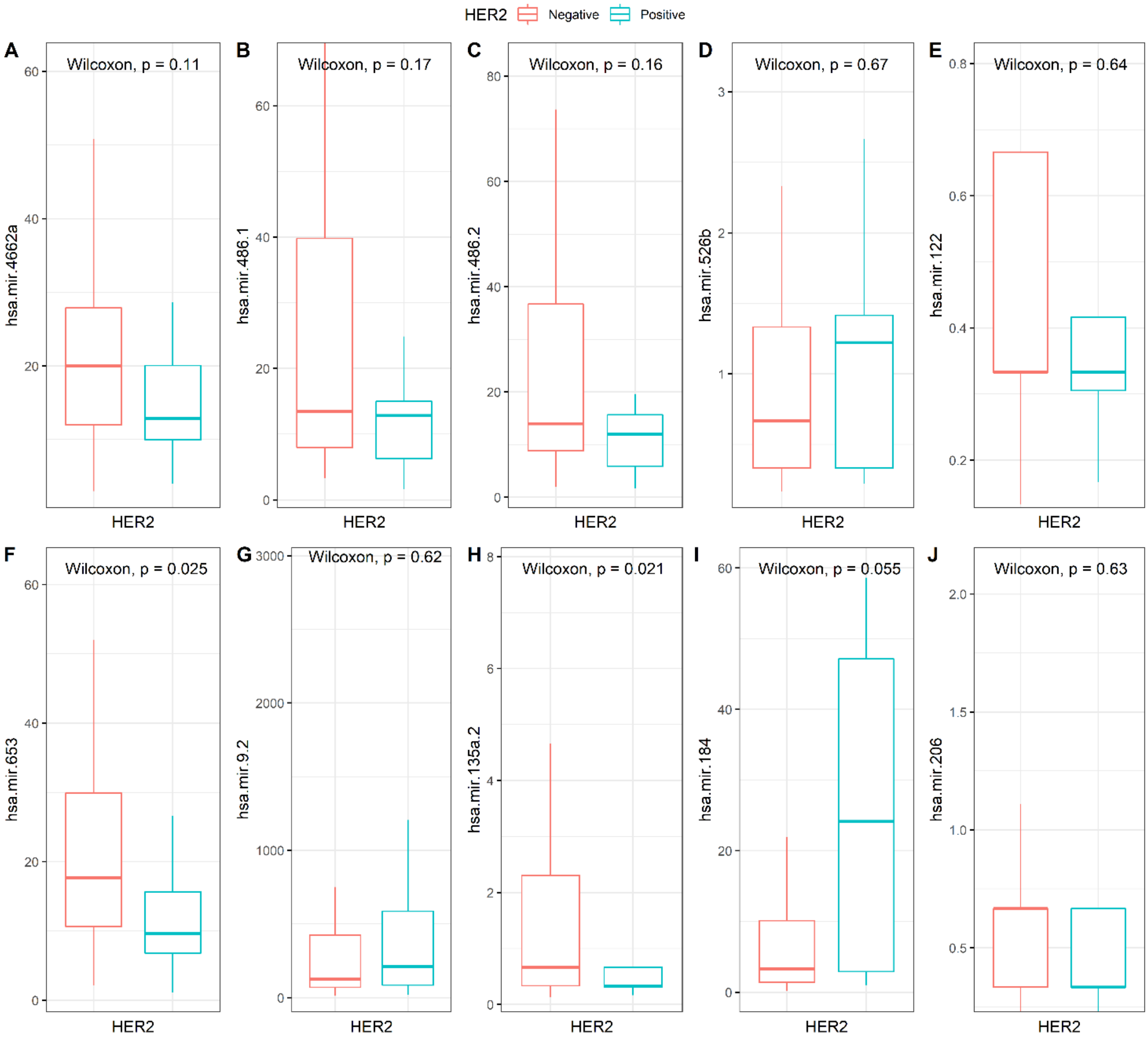

Figure S3: Relationship between breast cancer miRNAs expression and HER2 receptor status. (A-J) Extracted breast cancer miRNAs expression with associated p-values calculated using Wilcoxon sign rank test.

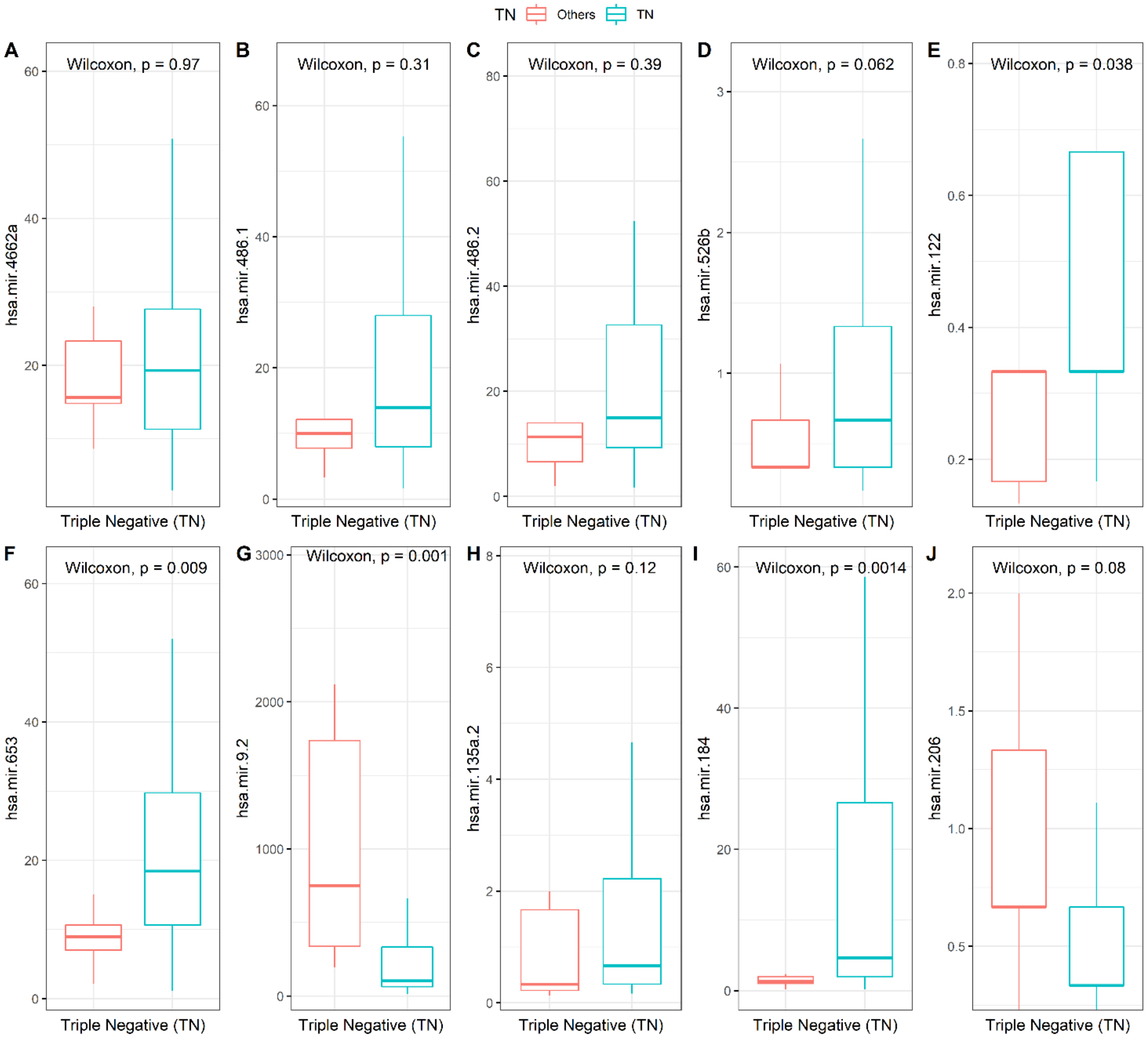

Figure S4: Relationship between breast cancer miRNAs expression and TN cases. (A-J) Extracted breast cancer miRNAs expression with associated p-values calculated using Wilcoxon sign rank test.

Table S4. Imaging-genomic associations. Spearman's correlations between the statistically significant radiomic features and miRNAs differentially expressed in breast cancer. Correlation between MRI radiomic features and miRNAs within Spearman's rho threshold ± 0.5. p value adjusted (Bonferroni correction) for statistical significance.

**ER negative**	**Features (UQ)**	**p-value adjusted**
hsa.mir.526b	G3	0.011
hsa.mir.653	S3	ns
hsa.mir.9.2	T5	ns
hsa.mir.206	S3	ns
**ER negative**	**Features (WHT)**	**p-value adjusted**
hsa.mir.9.2	G3	0.008
**PR negative**	**Features (UQ)**	**p-value adjusted**
hsa.mir.9.2	T5	0.036
hsa.mir.9.2	T6	0.023
**PR negative**	**Features (WHT)**	**p-value adjusted**
hsa.mir.135a.2	T5	0.010
hsa.mir.184	T2	0.026
hsa.mir.206	T2	0.006
**HER2 positive**	**Features (WHT)**	**p-value adjusted**
hsa.mir.486.2	M3	ns
**Triple negative**	**Features (UQ)**	**p-value adjusted**
hsa.mir.653	G2, S3	ns
hsa.mir.9.2	E2	ns
hsa.mir.206	G2, S3	ns
**Triple negative**	**Features (WHT)**	**p-value adjusted**
hsa.mir.486.1	G2, S2	ns
hsa.mir.486.2	G2, S2	ns, 0.021
hsa.mir.526b	G2	0.004
hsa.mir.653	S2	ns
hsa.mir.9.2	E2, T6, T11	ns, 0.04, ns
hsa.mir.206	S2	0.002

Table S5. Support Vector Machine Performance on Testing dataset ER+ Vs ER-

Feature Names	T5, T11	T5, T11	T5, T11	T5, T11	T5, G3	T5, T11	T5, T11, G3	T11, S2
Normalization Methods	NO	Scaling	Z-score	Robust Z-score	LOG	Upper Quartile	Quantile	WHT
Sensitivity	67%	67%	67%	67%	63%	67%	50%	77%
Specificity	83%	83%	83%	83%	50%	83%	83%	67%
Accuracy	75%	75%	75%	75%	57%	75%	67%	72%
AUC	85%	85%	85%	85%	68%	85%	71%	75%

NO: non-normalized features; LOG transformation method; WHT: Whitening normalization method.

Table S6. Random Forest Performance on Testing dataset ER+ Vs ER-

Feature Names	T5, S1	T5, S1	T5, S1	T5, S1	T5, S1	T5, S1	T5, T11, S1	T11, S2
Normalization Methods	NO	Scaling	Z-score	Robust Z-score	LOG	Upper Quartile	Quantile	WHT
Sensitivity	67%	67%	67%	67%	67%	67%	73%	77%
Specificity	83%	83%	83%	83%	83%	83%	83%	83%
Accuracy	75%	75%	75%	75%	75%	75%	78%	80%
AUC	83%	83%	83%	83%	85%	83%	83%	86%

NO: non-normalized features; LOG transformation method; WHT: Whitening normalization method.

Table S7. Naïve Bayesian Performance on Testing dataset ER+ Vs ER-

Feature Names	T5, T11, S1, S3	T5, S2, G3	T5, S3, G3	T5, S3, G3	T5, T11, S1, S2	T5, T11, S1, S3	T5, S1	T11, S2
Normalization Methods	NO	Scaling	Z-score	Robust Z-score	LOG	Upper Quartile	Quantile	WHT
Sensitivity	70%	70%	63%	63%	73%	70%	63%	77%
Specificity	67%	83%	83%	83%	67%	67%	83%	67%
Accuracy	68%	77%	73%	73%	70%	68%	73%	72%
AUC	84%	86%	86%	86%	83%	84%	80%	70%

NO: non-normalized features; LOG transformation method; WHT: Whitening normalization method.

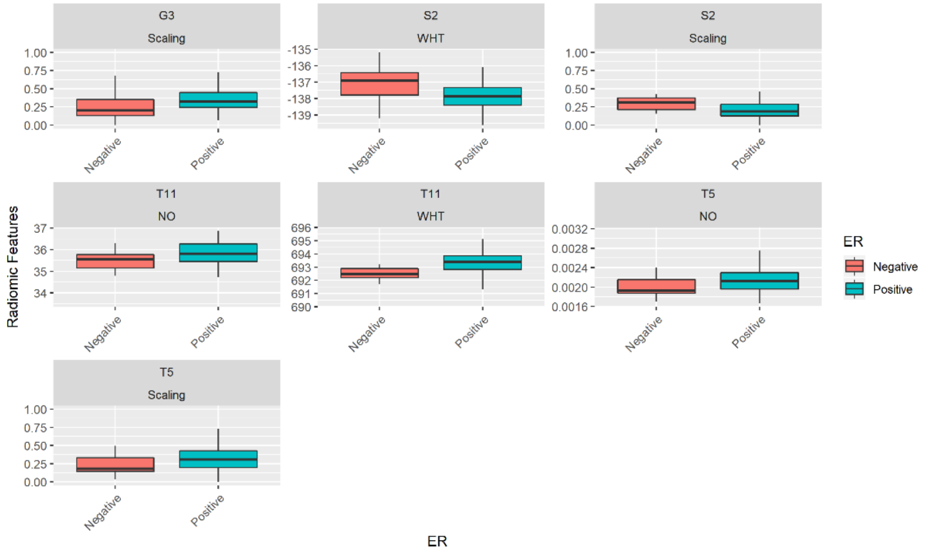

Figure S5. Box plots of radiomic features chosen by the machine learning methods to automatically detect ER status.

Table S8. Support Vector Machine Performance on Testing dataset PR+ Vs PR-

Feature Names	E3, T5	E3, T5	E3, T5	E3, T5	E3, T5	E3, T5	E3, T5	T2, T5
Normalization Methods	NO	Scaling	Z-score	Robust Z-score	LOG	Upper Quartile	Quantile	WHT
Sensitivity	61%	61%	61%	61%	61%	61%	61%	82%
Specificity	88%	88%	88%	88%	88%	88%	88%	50%
Accuracy	74%	74%	74%	74%	74%	74%	74%	66%
AUC	79%	79%	79%	79%	79%	79%	84%	73%

NO: non-normalized features; LOG transformation method; WHT: Whitening normalization method.

Table S9. Random Forest Performance on Testing dataset PR+ Vs PR-

Feature Names	E3, T4	E3, T4	E3, T4	E3, T4	E3, T4	E3, T4	E3, T5	T2, S2
Normalization Methods	NO	Scaling	Z-score	Robust Z-score	LOG	Upper Quartile	Quantile	WHT
Sensitivity	61%	61%	61%	61%	64%	61%	68%	61%
Specificity	75%	75%	75%	75%	75%	75%	75%	63%
Accuracy	68%	68%	68%	68%	70%	68%	71%	62%
AUC	71%	71%	71%	71%	74%	71%	81%	69%

NO: non-normalized features; LOG transformation method; WHT: Whitening normalization method.

Table S10. Naïve Bayesian Performance on Testing dataset PR+ Vs PR-

Feature Names	E4, T5, T6	E4, T5, T6	E4, T5, T6	E4, T5, T6	E4, T5, T6	E4, T5, T6	E4, T5	T2, T5
Normalization Methods	NO	Scaling	Z-score	Robust Z-score	LOG	Upper Quartile	Quantile	WHT
Sensitivity	61%	64%	57%	57%	64%	61%	71%	75%
Specificity	88%	88%	88%	88%	88%	88%	99%	50%
Accuracy	74%	76%	72%	72%	76%	74%	86%	63%
AUC	85%	85%	86%	86%	85%	85%	93%	77%

NO: non-normalized features; LOG transformation method; WHT: Whitening normalization method.

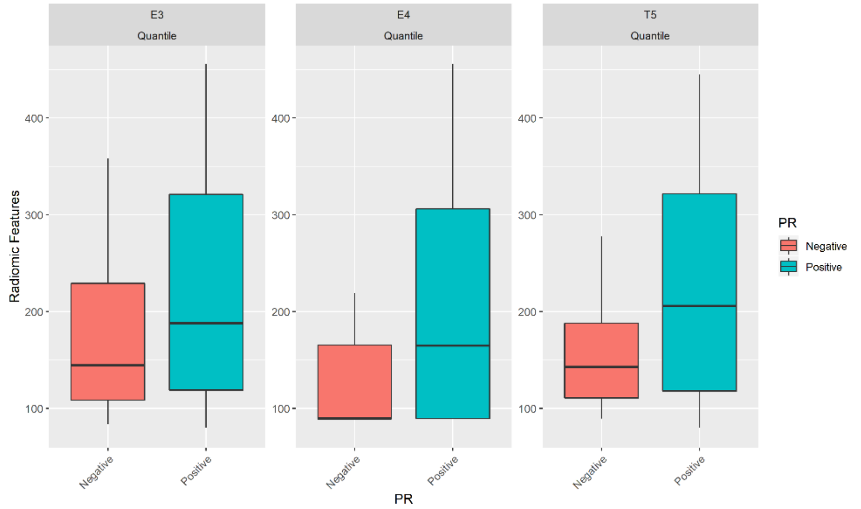

Figure S6. Box plots of radiomic features chosen by the machine learning methods to automatically detect PR status.

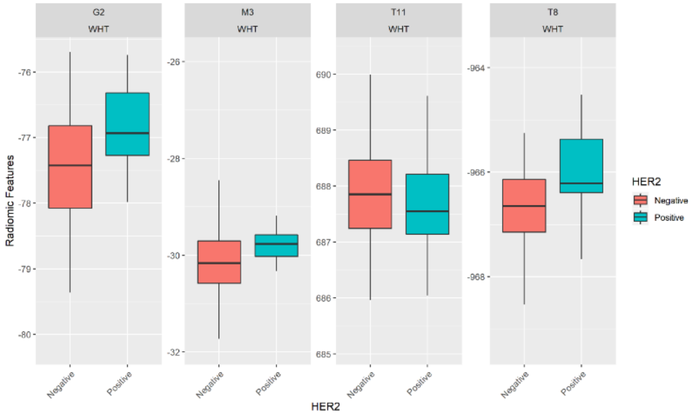

Figure S7. Box plots of radiomic features chosen by the machine learning methods to automatically detect HER2 receptor.

Table S11. Support Vector Machine Performance on Testing dataset TN Vs Others

Feature Names	E2, G2	E2, G2	E2, G2	E2, G2	E2, G2	E2, G2	E2, G2, S1, S3	E2, G2
Normalization Methods	NO	Scaling	Z-score	Robust Z-score	LOG	Upper Quartile	Quantile	WHT
Sensitivity	80%	80%	80%	80%	80%	80%	80%	80%
Specificity	50%	50%	50%	50%	57%	50%	53%	73%
Accuracy	65%	65%	65%	65%	68%	65%	67%	77%
AUC	80%	80%	80%	80%	82%	80%	74%	83%

NO: non-normalized features; LOG transformation method; WHT: Whitening normalization method.

Table S12. Random Forest Performance on Testing dataset TN Vs Others

Feature Names	E2, S2	E2, S2	E2, S2	E2, S2	G2, S1, S2	E2, S2	E2, G2	T11, G2
Normalization Methods	NO	Scaling	Z-score	Robust Z-score	LOG	Upper Quartile	Quantile	WHT
Sensitivity	50%	50%	50%	50%	50%	50%	50%	98%
Specificity	72%	72%	72%	72%	69%	72%	76%	76%
Accuracy	61%	61%	61%	61%	60%	61%	63%	88%
AUC	79%	79%	79%	79%	74%	79%	74%	91%

NO: non-normalized features; LOG transformation method; WHT: Whitening normalization method.

Table S13. Naïve Bayesian Performance on Testing dataset TN Vs Others

Feature Names	E2, S1, S2, S3	E2, S3	E2, G2, S1	E2, G2, S1	E2, S1, S2	E2, S1, S2, S3	E2, G2, S1	T11, S2
Normalization Methods	NO	Scaling	Z-score	Robust Z-score	LOG	Upper Quartile	Quantile	WHT
Sensitivity	50%	50%	20%	20%	75%	50%	55%	75%
Specificity	72%	79%	90%	90%	55%	72%	62%	66%
Accuracy	61%	65%	45%	45%	65%	61%	61%	70%
AUC	70%	67%	66%	66%	71%	70%	60%	77%

NO: non-normalized features; LOG transformation method; WHT: Whitening normalization method.

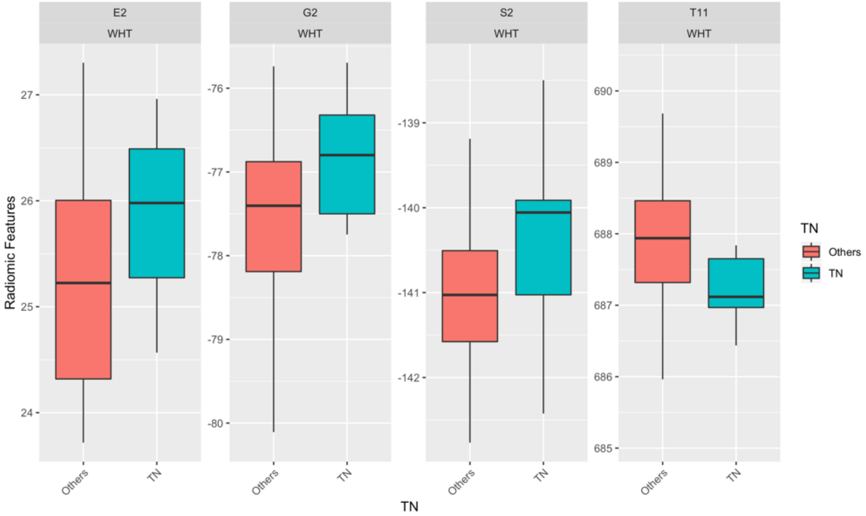

Figure S8. Box plots of radiomic features chosen by the machine learning methods to automatically detect TN cases.
